# Food purchasing places classification system based on the Dietary Guidelines for the Brazilian Population: Locais-Nova

**DOI:** 10.1590/S2237-96222025v34.20240361.en

**Published:** 2025-04-07

**Authors:** Marcos Anderson Lucas da Silva, Larissa Loures Mendes, Maria Alvim Leite, Luana Lara Rocha, Camila Aparecida Borges, Renata Bertazzi Levy, Maria Laura da Costa Louzada

**Affiliations:** 1 Universidade de São Paulo, Faculdade de Saúde Pública, Programa de Pós-Graduação em Nutrição em Saúde Pública, São Paulo, SP, Brazil; 2 Universidade de São Paulo, Núcleo de Pesquisas Epidemiológicas em Nutrição e Saúde, São Paulo, SP, Brazil; 3 Universidade Federal de Minas Gerais, Escola de Enfermagem, Programa de Pós-graduação em Saúde Pública, Belo Horizonte, MG, Brazil; 4 Universidade Federal de Minas Gerais, Grupo de Estudos, Pesquisas e Práticas em Ambiente Alimentar e Saúde, Belo Horizonte, MG, Brazil; 5 Universidade de São Paulo, Faculdade de Medicina, Departamento de Medicina Preventiva, São Paulo, SP, Brazil; 6 Universidade de São Paulo, Escola Superior de Agricultura Luiz de Queiroz, Departamento de Ciência e Tecnologia de Alimentos, Piracicaba, SP, Brazil

**Keywords:** Food Guides, Primary Health Care, Places of Purchase, Nova Classification, Food Acquisition, Guías Alimentarias, Atención Primaria de Salud, Lugares de Compra, Clasificación Nova, Adquisición de Alimentos

## Abstract

**Objective:**

To propose a new classification system for food purchasing places (Locais-Nova) based on the *Dietary Guidelines for the Brazilian Population*.

**Methods:**

We used 2017–2018 Brazilian Household Budgets Survey data on household food purchasing. Foods were categorized, according to the Nova classification, into unprocessed or minimally processed food, processed culinary ingredients, processed foods and ultra-processed foods. We estimated the average share of each Nova classification group in the total of grams acquired in Brazil. This estimate was compared with the average share of each Nova classification group in each of the 16 purchasing places assessed. Places were classified as “purchasing sources” for a specific Nova classification group whenever that group’s share a given place was equal to or greater than the national average.

**Results:**

Locais-Nova identified three categories of purchasing places: sources of unprocessed or minimally processed food and processed culinary ingredients, sources of processed foods and sources of ultra-processed foods. Fruits, vegetables, and farm products and butcher shops stood out as the main sources of unprocessed or minimally processed food; minimarkets and grocery stores were the main sources of ultra-processed foods; and bakeries and confectionaries, stood out as sources of processed and ultra-processed foods. Supermarkets were classified as sources of unprocessed or minimally processed food and ultra-processed foods.

**Conclusion:**

This study presented an innovative classification of food purchasing places. This reflected the recommendations of the *Dietary Guidelines for the Brazilian Population* and made it possible to understand food purchasing patterns in different types of purchasing places.

## Resumen

**Objetivo:**

Proponer un nuevo sistema de clasificación de lugares de adquisición de alimentos basado en la *Guía Alimentaria para la Población Brasileña*, denominado Locais-Nova.

**Métodos:**

Este estudio utilizó datos de la Encuesta de Presupuesto Familiar 2017-2018 sobre la adquisición familiar de alimentos. Los alimentos se categorizaron, según la clasificación Nova, en alimentos naturales o mínimamente procesados, ingredientes culinarios, alimentos procesados ​​y alimentos ultraprocesados. Se estimó la contribución promedio de cada grupo de la clasificación Nova al total de gramos adquiridos en Brasil. Esta estimación se comparó con la contribución promedio de cada grupo de clasificación Nova en cada uno de los 16 lugares de adquisición evaluados. Los lugares se clasificaron como “fuentes de adquisición” para un nuevo grupo de clasificación específico siempre que la contribución de ese grupo en un lugar determinado fuera igual o mayor que el promedio nacional.

**Resultados:**

Locais-Nova identificó tres categorías de lugares de compra: fuentes de alimentos e ingredientes culinarios frescos o mínimamente procesados, fuentes de alimentos procesados ​​y fuentes de alimentos ultraprocesados. Las fruterías y carnicerías se destacaron como principales fuentes de alimentos frescos o mínimamente procesados; minimercados y tiendas de abarrotes, como principales fuentes de alimentos ultraprocesados; y panaderías y confiterías, como fuentes de alimentos procesados ​​y ultraprocesados. Los supermercados se clasificaron como fuentes de alimentos frescos o mínimamente procesados ​​y ultraprocesados.

**Conclusión:**

Este estudio presentó una clasificación innovadora de los lugares de adquisición de alimentos. Esto reflejó las recomendaciones de la *Guía Alimentaria para la Población Brasileña* y permitió comprender patrones de adquisición de alimentos en los diferentes tipos de lugares.

## Introduction

The *Dietary Guidelines for the Brazilian Population* was published in 2014 by the Ministry of Health of Brazil. It recommends that diets be based on unprocessed or minimally processed food and processed culinary ingredients and that ultra-processed foods be avoided (1). Evidence shows that the population has been moving away from these recommendations. The caloric share of ultra-processed foods increased from 14.3%, in 2002-2003, to 19.4%, in 2017-2018. In the same period, unprocessed or minimally processed foods consumption went down from 51.0% to 48.7%, and unprocessed or minimally processed food and processed culinary ingredients consumption fell from 25.5% to 21.6% (2). 

There are potential connections between this transition in dietary patterns and recent changes in food access structures (3), including in retail environments. The places where people purchase food can influence food consumption through characteristics such as location and geographic accessibility and product variety, quality, price and advertising strategies (4,5). The Dietary Guidelines highlights as obstacles to healthy eating the ubiquity of food purchasing places that sell mostly ultra-processed foods, usually accompanied by advertisements, and difficulty in accessing places that sell unprocessed or minimally processed foods, particularly perishable foods, such as fruit, greens and vegetables (1). 

In Brazil, changes can be seen in the patterns of food purchasing places. Between 2003 and 2009, supermarket share of total purchases increased from 49.0% (6) to 59.0% (7). In parallel, purchases in more traditional places, such as open-air markets, have decreased (6,7), with a lower density of places selling healthy foods in some territories in Brazil (8-10).

Food purchasing places can be influenced by public policies and regulations that promote or restrict access to certain foods. Incorporating analysis of the dynamics of these places into food and nutritional surveillance actions can be an important strategy for monitoring healthy eating obstacles and facilitators. This can significantly contribute to the formulation and implementation of actions aimed at reducing inequalities in access to quality food (11). In Primary Health Care, for example, it is increasingly recommended that health professionals should be familiar with the places where food is produced, sold and distributed, as this knowledge is fundamental for informing health care practices aligned with the reality of individuals, communities and their territories (11).

In recent decades, conceptual models and metrics have been proposed to characterize and evaluate the quality of retail environments (12-18). In Brazil, in 2018, the Interministerial Chamber of Food and Nutritional Security presented a national methodology, with the objective of understanding the territorial distribution of limited access to healthy foods throughout the country (19). Some weaknesses in this classification have been highlighted (9,20,21). For example, purchasing places were categorized as “unprocessed food purchasing establishments” or “ultra-processed food purchasing establishments” when more than 50.0% of the foods purchased belonged to these groups (19), although there was no clear basis for choosing this criterion. “Mixed establishments” were those “where there is a predominance of purchasing culinary preparations or processed foods or where there is no predominance of purchasing unprocessed or minimally processed foods or ultra-processed foods” (19). These establishments included places that sold both healthy and unhealthy foods, making it difficult to effectively monitor food purchasing environments.

This article aims to propose a new classification of food purchasing places based on the recommendations of the *Dietary Guidelines for the Brazilian Population*, called Locais-Nova. The proposal aims to provide a new reference that is useful in categorizing food purchasing places and that informs food and nutritional surveillance of determinants of healthy eating.

## Methods

### Data source and measurement

We used the 2017–2018 Brazilian Family Budget Survey data on household food acquisition. The Survey was conducted by the Brazilian Institute of Geography and Statistics (22) between July 2017 and July 2018.

The scope of the Survey was national, representative of the following domains: Brazil as a whole, its five major Regions (North, Northeast, Midwest, Southeast and South), urban areas and rural areas, the country’s 26 Federative Units and Federal District. A complex sampling plan was used, using clusters in two stages, randomly selecting census tracts in the first stage and households in the second stage. The census tracts were derived from a master sample provided by the Brazilian Institute of Geography and Statistics, grouped into household strata with high geographic and socioeconomic homogeneity (22).

The information analyzed referred to food for home consumption purchased by each consumption unit (household) for seven consecutive days. These purchases were recorded by household residents, or by an interviewer from the institute, in the household members’ expense book, considering monetary and non-monetary purchases, name of the item, quantity purchased (kilograms or liters) and places where the food was purchased (22).

The study units were comprised of clusters of households generated through the sampling plan (strata). In 2017-2018, the 57,920 households studied resulted in 575 strata with an average of 86.50 households (ranging from 16 to 524 per stratum). In this article, only information on monetary purchases was included.

### Food classification

Foods were categorized according to the Nova classification (23). The *Dietary Guidelines* e recommendations (1) were used as a basis, dividing foods into four groups according to their industrial processing characteristics: unprocessed or minimally processed foods (rice, beans, vegetables) – G1; processed culinary ingredients (salt, sugar) – G2; processed foods (cheese, bread) – G3; and ultra-processed foods (soft drinks, savory snacks) – G4 (23).

The unprocessed or minimally processed foods and processed culinary ingredients groups were assessed together in a single group (G1+G2). This grouping was aligned with the Guide’s golden rule (“always prefer unprocessed or minimally processed foods and culinary preparations instead of ultra-processed foods”) (1). 

Purchased food quantities were expressed as the average percentage of the share of each food group in relation to the total grams purchased. Correction factors were used to estimate the part of the food that could be consumed, excluding inedible parts (such as peel or bones) (24). Alcoholic beverages were excluded. 

### Food purchasing place grouping

The food purchasing places cited in the 2017-2018 Brazilian Family Budget Survey (n=296) were grouped by similarity based on the technical study entitled Mapping Food Deserts in Brazil (19). That study used as a reference the National Classification of Economic Activities, officially adopted by federal bodies that manage administrative records, to standardize the classification of establishments according to their main economic activity (19). Sixteen groups were created, selecting only places where purchases were made with money: supermarkets; minimarkets and grocery stores; fruits, vegetables, and farm products; bakeries and confectionaries; butcher shops; food street vendors; snack bars; bars; restaurants; convenience stores; fish markets; candy stores; dairy and cold cuts retailers; canteens in institutional environments; frozen and ready-to-eat food vendors; and other places (Supplementary Box 1). 

### Criteria for classifying food purchasing places as per the *Dietary Guidelines for the Brazilian Population*: **Locais-Nova classification system**

**Figure 1:**
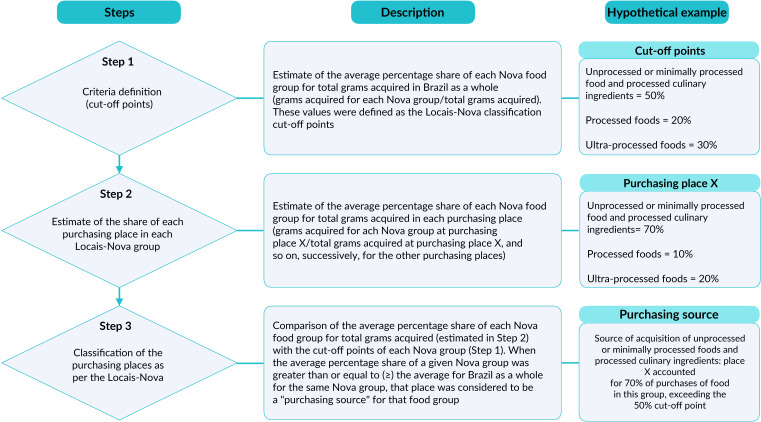
Steps for creating the Locais-Nova classification system criteria

The Locais-Nova (‘Locais’ refers to food purchasing places in Portuguese) classification system aims to classify food purchasing places according to the *Dietary Guidelines for the Brazilian population*. The Locais-Nova criteria (cutoff points) were established based on the average share of each Nova classification food group in relation to total grams purchased in Brazil. The average percentage share of Nova classification food groups was estimated in total grams acquired at each purchasing place. The average percentage of the share of each Nova Classification food group at each acquisition place was compared with the criteria established. When the average percentage share of food groups, according to the Nova classification, in a given purchasing place, was equal to or greater than the defined criteria, that place was classified as a “purchasing source” for that food group (Figure 1).

In some cases, a single purchasing place could be a source of two food groups. This happened when the average percentage share for that place was equal to or greater than the average of the criteria for two food groups. 

The three categories of the Locais-Nova classification system were: source of acquisition of unprocessed or minimally processed foods, source of acquisition of processed foods and processed culinary ingredients and source of acquisition of ultra-processed foods. Criteria were created, using the same methodology, for the five Brazilian Regions.

Five indicators were created to identify food purchasing places according to the Dietary Guidelines recommendations.

- Places for purchasing food that should be the basis of diets: source of acquisition of unprocessed or minimally processed foods and processed culinary ingredients.
- Places for purchasing food identified only as sources of foods that should form the basis of the diets: source of acquisition of only unprocessed or minimally processed foods and culinary ingredients.
- Places for purchasing food that should be limited: source of acquisition of processed foods
- Places for purchasing food that should be avoided: source of acquisition of ultra-processed foods.
- Places for purchasing food identified only as sources of food that should be avoided: sources of acquisition only of ultra-processed foods.

In the results we present point estimates (averages) of the percentage share of food groups in each purchasing place in Brazil as a whole and in its major Regions. 95% confidence intervals are presented in Supplementary Tables 2 to 7.

Weighting factors were applied depending on the sampling structure of the database. We used STATA 18/SE software to perform the analyses.

## Results

**Figure 2:**
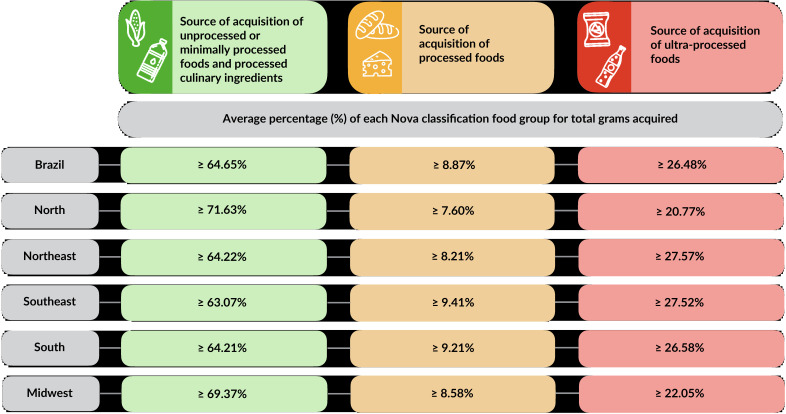
Criteria for classifying food purchasing places. Brazil and its Regions, 2017-2018

In Brazil, the cutoff points of the Locais-Nova classification system were: ≥64.65% of total grams for places that were purchasing sources of unprocessed or minimally processed food and processed culinary ingredients; ≥8.87% for places that were sources of processed foods; and ≥26.48% for locations that were sources of ultra-processed foods (Figure 2)

In the major Regions, differences were found in the criteria. The regions with the highest cut-off points for unprocessed or minimally processed food and processed culinary ingredients were the North (≥71.63%) and the Midwest (≥69.37%). The Southeast had the highest cutoff point for processed foods (≥9.21%). The Northeast had the highest cutoff point for ultra-processed foods (≥27.57%) (Figure 2).

In Brazil as a whole, 5 of the 16 purchasing places were sources only of unprocessed or minimally processed foods and processed culinary ingredients (fruits, vegetables, and farm products, butcher shops, food street vendors, fish markets and restaurants), 1 place was a source only of processed foods (dairy and cold cuts retailers) and 5 were sources only of ultra-processed foods (small markets and grocery stores, snack bars, candy stores, frozen and ready-to-eat food outlets, and other places) (Table 1). Five sites were sources of 2 groups. Among these, only supermarkets were a source of both unprocessed or minimally processed food and processed culinary preparations and ultra-processed foods. The others (bakeries and confectionaries, bars, convenience stores and canteens) were sources of processed foods and ultra-processed foods (Table 1).

**Table 1:** Average percentage (%) share of each Nova classification food group in total grams acquired at each purchasing place. Brazil, 2017-2018 (n=57,920)

Food purchasing places	G1+G2^a^	G3^b^	G4^c^
**Criteria for classifying food source purchasing places**	≥64.65	≥8.87	≥26.48
Supermarkets	69.29	3.67	27.04
Small markets and grocery stores	64.49	4.93	30.58
Fruits, vegetables, and farm products	93.69	1.81	4.50
Bakeries and confectionaries	18.28	54.90	26.82
Butcher shops	87.75	2.65	9.60
Food street vendors	74.76	8.72	16.52
Snack bars	11.57	0.89	87.54
Restaurants	84.08	0.81	15.11
Frozen and ready-to-eat food vendors	43.23	2.77	54.00
Bars	26.17	9.37	64.46
Convenience stores	7.32	13.63	79.05
Fish markets	99.65	0.35	0.00
Candy stores	9.54	3.80	86.66
Airy and cold cuts retailers	32.12	49.05	18.83
Canteens	44.96	11.62	43.42
Others	46.74	3.53	49.73

**Legend:** Source of acquisition of unprocessed or minimally processed foods and processed culinary ingredients Source of acquisition of processed foods Source of acquisition of ultra-processed foods ^a^G1+G2=unprocessed or minimally processed food (G1) and processed culinary ingredients (G2); ^b^G3=processed foods; ^c^G4=ultra-processed foods.

In all regions, the local sources of unprocessed or minimally processed food and processed culinary preparations were supermarkets, fruits, vegetables, and farm products, butcher shops, food street vendors, restaurants and fish markets (Table 2). Regional diversity was evident regarding the places that were sources of processed foods. In the South, there were fewer places that were sources of this food group (bakeries and confectionaries and dairy and cold cuts retailers). In the North, they were bakeries and confectionaries, bars and dairy and cold cuts retailers. In the Northeast, in addition to the places highlighted in the North, canteens were also included. In the Southeast, the places were bakeries and confectionaries, food street vendors, bars, convenience stores, dairy and cold cuts retailers, and canteens. In the Midwest, the places were Bakeries and confectionaries, food street vendors, convenience stores and dairy and cold cuts retailers (Table 2).

**Table 2:** Average percentage (%) share of each Nova classification food group in total grams acquired at each purchasing place, by Brazilian regions. Brazil, 2017-2018 (n=57,920)

Food purchasing places	North			Northeast			Southeast			South			Midwest		
	G1+G2^a^	G3^b^	G4^c^	G1+G2^a^	G3^b^	G4^c^	G1+G2^a^	G3^b^	G4^c^	G1+G2^a^	G3^b^	G4^c^	G1+G2^a^	G3^b^	G4^c^
**Criteria for classifying food source purchasing places** – **by region**	≥71.63	≥7.60	≥20.77	≥64.22	≥8.21	≥27.57	≥63.07	≥9.41	≥27.52	≥64.21	≥9.21	≥26.58	≥69.37	≥8.58	≥22.05
Supermarkets	75.73	3.18	21.09	72.70	3.58	23.72	67.77	3.75	28.48	67.12	4.31	28.57	73.96	3.15	22.89
Small markets and grocery stores	71.53	3.55	24.92	63.22	4.42	32.36	62.30	5.47	32.23	64.17	4.70	31.13	69.30	4.88	25.82
Fruits, vegetables, and farm products	95.81	1.24	2.95	96.23	1.59	2.18	93.23	1.29	5.48	89.94	3.72	6.34	93.10	2.11	4.79
Bakeries and confectionaries	11.39	66.76	21.85	14.49	67.20	18.31	23.63	50.43	25.94	18.18	44.29	37.53	20.44	49.29	30.27
Butcher shops	95.87	1.51	2.60	92.58	3.94	3.48	84.09	2.43	13.47	85.09	1.42	13.48	89.24	2.57	8.19
Food street vendors	80.86	3.65	15.49	75.51	9.29	15.20	69.96	10.69	19.35	80.96	4.42	14.62	81.84	8.77	9.39
Snack bars	35.38	0.59	64.03	11.86	2.09	86.05	9.72	0.43	89.85	8.79	0.02	91.19	6.08	1.83	92.09
Restaurants	90.19	0.71	9.10	86.11	0.99	12.90	81.07	0.96	17.97	89.96	0.29	9.75	77.53	0.59	21.87
Frozen and ready-to-eat food vendors	46.01	0.00	53.99	29.50	7.27	63.23	51.86	1.95	46.19	33.45	0.17	66.38	52.76	0.00	47.24
Bars	52.06	11.76	36.18	30.49	4.63	64.88	24.1	12.03	63.87	14.29	8.75	76.96	10.90	2.19	86.91
Convenience stores	0.00	0.17	99.83	13.32	7.05	79.63	1.28	21.39	77.33	17.23	6.06	76.71	11.17	10.82	78.01
Fish markets	100.00	0.00	0.00	98.88	1.12	0.00	100.00	0.00	0.00	100.00	0.00	0.00	100.00	0.00	0.00
Candy stores	0.00	4.19	95.81	16.65	6.86	76.49	7.42	4.05	88.53	16.51	0.00	83.49	0.00	0.00	100.00
Dairy and cold cuts retailers	34.85	31.66	33.49	49.32	38.15	12.53	21.02	54.46	24.52	38.59	60.19	1.22	8.52	45.06	46.42
Canteens	56.02	0.00	43.98	58.65	8.38	32.97	22.87	20.66	56.47	86.24	0.00	13.76	52.17	0.40	47.43
Others	49.07	1.68	49.25	57.42	3.51	39.07	36.78	2.59	60.63	59.90	7.84	38.26	53.82	1.65	44.53

Legend: Source of acquisition of unprocessed or minimally processed foods and processed culinary ingredients Source of acquisition of processed foods Source of acquisition of ultra-processed foods ^a^G1+G2=unprocessed or minimally processed food (G1) and processed culinary ingredients (G2); ^b^G3=processed foods; ^c^G4=ultra-processed foods.

In all regions, supermarkets, small markets and grocery stores, bakeries and confectionaries, snack bars, bars, convenience stores, candy stores, frozen and ready-to-eat food vendorss and other places were sources of ultra-processed foods. In the North and Midwest, dairy and cold cuts retailers were also included in this category (Table 2).

Although all places were classified according to the Locais-Nova criteria based on estimated averages, places with a low share in purchases, such as canteens throughout Brazil, bars and dairy and cold cuts retailers” in the North, canteens in the Northeast, and bars and convenience stores in the Southeast and Midwest presented wide confidence intervals (95%CI), making their classification uncertain.

Fruits, vegetables, and farm products, butcher shops, restaurants and fish markets appeared in all regions of the country as places that were exclusive for purchasing food sources that should be the basis of diets (Box 1). The Northeast was the only region that included supermarkets in this category, while in the South, canteens were included. Small markets and grocery stores, snack bars and candy stores appeared in all regions as places that are exclusively sources of foods that should be avoided. In the North and Midwest, canteens also appeared. In the Northeast, bars also appeared (Box 1). 

**Box 1:** Food purchasing places as per the recommendations of the Dietary Guidelines for the Brazilian population. Brazil and Brazilian Regions, 2017-2018 (n=57,920)

Indicators	Brazil	North	Northeast	Southeast	South	Midwest
Places for purchasing food sources that should be the basis of diets - source of acquisition of unprocessed or minimally processed foods and processed culinary ingredients	Supermarkets Fruits, vegetables, and farm products Butcher shops Food street vendors Restaurants Fish markets	Supermarkets Fruits, vegetables, and farm products Butcher shops Food street vendors Restaurants Fish markets	Supermarkets Fruits, vegetables, and farm products Butcher shops Food street vendors Restaurants Fish markets	Supermarkets Fruits, vegetables, and farm products Butcher shops Food street vendors Restaurants Fish markets	Supermarkets Fruits, vegetables, and farm products Butcher shops Food street vendors Restaurants Fish markets Canteens	Supermarkets Fruits, vegetables, and farm products Butcher shops Food street vendors Restaurants Fish markets
Purchasing places identified only as a source of food that should be the basis of diets - source of acquisition of only unprocessed or minimally processed foods and culinary ingredients	Fruits, vegetables, and farm products Butcher shops Food street vendors Fish markets Restaurants	Fruits, vegetables, and farm products Butcher shops Food street vendors Restaurants Fish markets	Supermarkets Fruits, vegetables, and farm products Butcher shops Restaurants Fish markets	Fruits, vegetables, and farm products Butcher shops Restaurants Fish markets	Fruits, vegetables, and farm products Butcher shops Food street vendors Fish markets Restaurants Canteens	Fruits, vegetables, and farm products Butcher shops Restaurants Fish markets
Places for purchasing food sources that should be limited - source of acquisition of processed foods	Bakeries and confectionaries Bars Convenience stores Dairy and cold cuts retailers Canteens	Bakeries and confectionaries Bars Dairy and cold cuts retailers	Bakeries and confectionaries Food street vendors Dairy and cold cuts retailers Canteens	Bakeries and confectionaries Food street vendors Bars Convenience stores Dairy and cold cuts retailers Canteens	Bakeries and confectionaries Dairy and cold cuts retailers	Bakeries and confectionaries Food street vendors Convenience stores Dairy and cold cuts retailers
Places for purchasing food sources that should be avoided - source of acquisition of ultra-processed foods	Supermarkets Small markets and grocery stores Bakeries and confectionaries Snack bars Bars Convenience stores Candy stores Canteens Frozen and ready-to-eat food vendors Others	Supermarkets Small markets and grocery stores Bakeries and confectionaries Snack bars Bars Convenience stores Candy stores Dairy and cold cuts retailers Canteens Frozen and ready-to-eat food vendors Others	Small markets and grocery stores Snack bars Bars Convenience stores Candy stores Canteens Frozen and ready-to-eat food vendors Others	Supermarkets Small markets and grocery stores Snack bars Bars Convenience stores Candy stores Canteens Frozen and ready-to-eat food vendors Others	Supermarkets Small markets and grocery stores Bakeries and confectionaries Snack bars Bars Convenience stores Candy stores Frozen and ready-to-eat food vendors Others	Supermarkets Small markets and grocery stores Bakeries and confectionaries Snack bars Bars Convenience stores Candy stores Dairy and cold cuts retailers Canteens Frozen and ready-to-eat food vendors Others
Purchasing places identified only as sources of food that should be avoided - sources of acquisition only of ultra-processed foods	Small markets and grocery stores Snack bars Candy stores Frozen and ready-to-eat food vendors Others	Small markets and grocery stores Snack bars Convenience stores Candy stores Canteens Frozen and ready-to-eat food vendors Others	Small markets and grocery stores Snack bars Bars Convenience stores Candy stores Frozen and ready-to-eat food vendors Others	Small markets and grocery stores Snack bars Candy stores Frozen and ready-to-eat food vendors Others	Small markets and grocery stores Snack bars Bars Candy stores Frozen and ready-to-eat food vendors Others	Small markets and grocery stores Snack bars Bars Candy stores Canteens Frozen and ready-to-eat food vendors Others

## Discussion

This study presented a new methodology for classifying food purchasing places according to the Dietary Guidelines. By analyzing groups of foods purchased in different categories of places, criteria were established capable of distinguishing between places that promote healthy eating and places that encourage unhealthy eating, considering the purchasing pattern in the different purchasing places in Brazil. Regional stratification allowed us to customize this classification for specific geographic contexts, providing more details about food environments. 

The economic and cultural particularities of each region influenced the characteristics of the environments and the dynamics of access to food (2,25). This explained why a purchase location was a source of a given group in one region, but not in another. Considering the places that, cumulatively, accounted for more than 90% of the share of the total purchased (Supplementary Table 1), we found that greengrocers and butcher shops were consistently identified as food sources that should be the basis of the Brazilian diet. Small markets and grocery stores were classified as places to avoid. Supermarkets were the only place identified as a source of unprocessed or minimally processed food and processed culinary ingredients and ultra-processed foods.

The food purchasing data showed that supermarkets were the most accessed food purchasing places in Brazil, followed by small markets and grocery stores, fruits, vegetables, and farm products and bakeries (6,7). Although supermarkets are a source of healthy foods, other evidence has indicated that using them as the sole purchasing source of healthy foods may not be the most appropriate strategy (26,27). This is because unprocessed or minimally processed foods often compete with the abundance of ultra-processed foods in this purchasing place, such as soft drinks, cookies, among others (1). Within these places, these foods are often promoted through aggressive advertising and attractive promotions (5,28,29), which can undermine healthier food choices.

Supermarkets are considered more convenient and, in terms of overall purchasing, have prices on average 37% lower compared to other purchasing places (30). Supermarkets being close to street markets that sell unprocessed food can have a negative impact. Evidence has shown that the presence of supermarkets close to these places led to a reduction in the prices of fruit and vegetables in supermarkets, in an attempt to compete with nearby fruits, vegetables, and farm products (31). When the number of fruits, vegetables, and farm products decreases, supermarkets may increase prices due to lack of competition, making access to healthy and diversified foods more difficult for the population.

We also created the indicator “Places for purchasing food identified only as sources of foods that should form the basis of the diets”, which includes places that, using the method proposed, were considered to be a source of unprocessed or minimally processed food and processed culinary preparations. This indicator was proposed as the metric that identifies places with food groups that should be the basis of diets. Fruits, vegetables, and farm products, butcher shops, fish markets and restaurants, identified as food purchasing places that should be the basis of nutrition, have the potential to play a crucial role in promoting healthy eating. 

The methodology presented in this article makes it possible to map access to food in Brazil with great scope and on a large scale. This allows us to observe the specificity of each place, where carrying out detailed audits would be unfeasible. This classification can be applied, for example, in databases recording geographic coordinates of food sales places. An example of these databases is the Annual Social Information List, an open access government database. It classifies purchasing places according to the National Classification of Economic Activities, which allows identification of different types of food purchasing places (32).

Regular application of the Locais-Nova classification system to food purchasing places distribution data will allow us to identify and monitor the transformations these places undergo. New purchasing patterns may emerge and certain places may become different food sources or remain unchanged. Such monitoring will allow the development of strategies to prevent places from becoming predominantly a source of ultra-processed foods, as well as prioritizing territories that need facilities that offer healthy foods. Among the possible measures, financial or tax incentives stand out for traders who increase the supply of healthy foods, such as fruit, vegetables and other unprocessed or minimally processed foods. Also noteworthy is the creation of new points of sale, such as street markets or public markets, especially in areas with low availability of healthy foods. 

Continuous updates to the classification system presented in this article may, as long as information sources exist, incorporate indicators that include the evaluation of additional characteristics of places, such as price, advertising and size of food purchasing places. The Locais-Nova system can be reproduced in other countries and contexts where databases on food acquisition are available, generating specific cutoff points for each territory.

There were some limitations, including the fact that the purchasing element of the Brazilian Household Budgets Survey did not consider the purchase of food for consumption outside the home. This is prejudicial, in particular, to the assessment of establishments such as canteens, restaurants, bars, snack bars, food street vendors and dairy and cold cuts retailers, which together accounted for less than 1% of the total share of purchases. Despite this, the majority of purchases were for consumption at home (33). The survey data related to 2017-2018 and the scenario may have changed significantly since then, especially in the post-COVID-19 pandemic period, due to the increase in online shopping (34,35). 

The 2017–2018 Brazilian Family Budget Survey is the most recent representative national survey and has a sufficient level of detail to identify the purchasing places and food groups studied. As soon as it is published the methodology will be able to be applied to new research. Using the Nova classification provides analysis of purchasing places based on the degree of food processing, a crucial paradigm for understanding food systems today. This study also presented methodology aligned with the scope of food and nutritional surveillance and in line with the Dietary Guidelines recommendations. 

In conclusion, a new classification system for food purchasing places was presented, based on the guidelines of the *Dietary Guidelines for the Brazilian Population*, to identify how different compositions of food environments influence consumption patterns. 

Regional disparities suggest the need for strategies adapted to local specificities. Including this methodology in future surveys will enable continuous and detailed monitoring of the food environment, supporting the formulation of public policies aimed at improving the health of the Brazilian population, focusing on retail food outlets.

## References

[1] Brasil (2014). Ministério da Saúde.

[2] Levy RB, Andrade GC, Cruz GL, Rauber F, Louzada MLC, Claro RM (2022). Três décadas da disponibilidade domiciliar de alimentos segundo a NOVA – Brasil, 1987-2018. Rev Saude Publica.

[3] Swinburn BA, Kraak VI, Allender S, Atkins VJ, Baker PI, Bogard JR (2019). The global syndemic of obesity, undernutrition, and climate change: the Lancet Commission report. Lancet.

[4] Caspi CE, Sorensen G, Subramanian SV, Kawachi I (2012). The local food environment and diet: a systematic review. Health Place.

[5] Borges CA, Gabe KT, Canella DS, Jaime PC (2021). Caracterização das barreiras e facilitadores para alimentação adequada e saudável no ambiente alimentar do consumidor. Cad. Saúde Pública.

[6] Costa JC, Claro RM, Martins APB, Levy RB (2013). Food purchasing sites. Repercussions for healthy eating. Appetite.

[7] Machado PP, Claro RM, Martins APB, Costa JC, Levy RB (2018). Is food store type associated with the consumption of ultra-processed food and drink products in Brazil?. Public Health Nutr.

[8] Grilo MF, Menezes C, Duran AC (2022). Food swamps in Campinas, Brazil. Cien Saude Colet.

[9] Borges DC, Vargas JCB, Honório OS, Mendes LL, Canuto R (2024). Social and ethnic-racial inequities in the occurrence of food deserts in a Brazilian state capital. Food Sec.

[10] Rocha LL, Friche AAL, Melo GBV, Cordeiro NG, Honório OS, Cardoso LO (2024). Food retail in favelas of a Brazilian metropolis. Food Sec.

[11] Brasil (2022). Matriz para organização dos cuidados em alimentação e nutrição na Atenção Primária à Saúde.

[12] Mendes LL, Rocha LL, Botelho LV, Menezes MC, Castro PCP, Camara AO (2023). Scientific research on food environments in Brazil: a scoping review. Public Health Nutr.

[13] Glanz K, Sallis JF, Saelens BE, Frank LD (2005). Healthy nutrition environments: concepts and measures. Am J Health Promot.

[14] Swinburn B, Sacks G, Vandevijvere S, Kumanyika S, Lobstein T, Neal B (2013). INFORMAS (International Network for Food and Obesity/non-communicable diseases Research, Monitoring and Action Support): overview and key principles. Obes Rev.

[15] Story M, Kaphingst KM, Brien R, Glanz K (2008). Creating healthy food and eating environments: policy and environmental approaches. Annu Rev Public Health.

[16] Borges CA, Jaime PC (2019). Desenvolvimento e avaliação de instrumento de auditoria do ambiente alimentar: AUDITNOVA. Rev Saude Publica.

[17] Downs SM, Ahmed S, Fanzo J, Herforth A (2020). Food environment typology: advancing an expanded definition, framework, and methodological approach for improved characterization of wild, cultivated, and built food environments toward sustainable diets. Foods.

[18] Bogard JR, Andrew NL, Farrell P, Herrero M, Sharp MK, Tutuo J (2021). A typology of food environments in the Pacific region and their relationship to diet quality in Solomon Islands. Foods.

[19] Câmara Interministerial de Segurança Alimentar e Nutricional (2018). Mapeamento dos desertos alimentares no Brasil: estudo técnico.

[20] Justiniano ICS, Menezes MC, Mendes LL, Pessoa MC (2022). Retail food environment in a Brazilian metropolis over the course of a decade: evidence of restricted availability of healthy foods. Public Health Nutr.

[21] Andretti B, Cardoso LO, Honório OS, Castro PCP, Tavares LF, Silva ICG (2023). Ecological study of the association between socioeconomic inequality andfood deserts and swamps around schools in Rio de Janeiro, Brazil. BMC Public Health.

[22] Instituto Brasileiro de Geografia e Estatística (2019). Pesquisa de Orçamentos Familiares, 2017-2018: primeiros resultados.

[23] Monteiro CA, Cannon G, Levy RB, Moubarac JC, Louzada ML, Rauber F (2019). Ultra-processed foods: what they are and how to identify them. Public Health Nutr.

[24] (2024). Tabela Brasileira de Composição de Alimentos (TBCA).

[25] Silva MAL, Louzada MLC, Levy RB (2022). Disponibilidade domiciliar de alimentos regionais no Brasil: distribuição e evolução 2002-2018. Segur Aliment Nutr.

[26] Honório OS, Horta PM, Pessoa MC, Jardim MZ, Carmo AS, Mendes LL (2022). Food deserts and food swamps in a Brazilian metropolis: comparison of methods to evaluate the community food environment in Belo Horizonte. Food Sec.

[27] Wagner J, Hinton L, McCordic C, Owuor S, Capron G, Arellano SG (2019). Do urban food deserts exist in the Global South? An analysis of Nairobi and Mexico City. Sustainability.

[28] Charlton EL, Kähkönen LA, Sacks G, Cameron AJ (2015). Supermarkets and unhealthy foodmarketing: an international comparison of the content of supermarket catalogues/circulars. Prev Med.

[29] Moreira CC, Silva ACF, Leme AOR, Silva TS, Brito FSB, Oliveira ASD (2023). Healthy eating in supermarket circulars: reflections according to the food classification adopted in dietary guidelines for the Brazilian population. Cienc Saude Colet.

[30] Machado PP, Claro RM, Canella DS, Sarti FM, Levy RB (2017). Price and convenience: the influence of supermarkets on consumption of ultra-processed foods and beverages in Brazil. Appetite.

[31] Valpiani N, Wilde P, Rogers B, Stewart H (2015). Patterns of fruit and vegetable availability and price competitiveness across four seasons are different in local food outlets and supermarkets. Public Health Nutr.

[32] Brasil (2024). Relação Anual de Informações Sociais.

[33] Bezerra IN, Moreira TMV, Cavalcante JB, Souza AM, Sichieri R (2017). Food consumed outside the home in Brazil according to places of purchase. Rev Saude Publica.

[34] Horta PM, Souza JPM, Rocha LL, Mendes LL (2021). Digital food environment of a Brazilian metropolis: food availability and marketing strategies used by delivery apps. Public Health Nutr.

[35] Downs SM, Ahmed S, Warne T, Fanzo J, Loucks K (2022). The global food environment transition based on the socio-demographic index. Global Food Security.

