# Supplementary material for: Food purchasing places classification system based on the Dietary Guidelines for the Brazilian Population: Locais-Nova
Source: Epidemiol Serv Saude. 2025 Apr 7;34:e20240361. doi: 10.1590/S2237-96222025v34.20240361.en (PMC11998909; doi:10.1590/S2237-96222025v34.20240361.en)
Supplement: Supplementary file 1 [file 2237-9622-ress-34-e20240361-supp01.pdf]

Quadro suplementar 1. Agrupamentos dos locais de aquisição de alimentos. Brasil, 2017-2018

**Grupos**	**Agrupamento detalhado**
Supermercados	Hipermercado, hipermercado atacadista, atacadista, loja atacadista, supermercado, supermercado atacadista, mercantil
Minimercados e mercearias	Mercearia, mercado (armazém), mercadinho (quitanda), bodega (mercearia), bodega (armazém), armazém, minimercado (armazém), mercado municipal, mercado estadual, mercadinho, empório, birosca, armazém varejista, secos e molhados (armazém), mercado popular público, mercado público popular, cerealista, venda, barracão, tendinha (quitanda), mini-box, casa de farinha, casa do norte, armazém atacadista, cerealista atacadista
Hortifrutigranjeiros	Hortifruti, loja de frutas, fruteira (frutaria), banca de frutas, feira de frutas, frutaria, verdureiro, feira de verduras, banca de verduras, casa de verduras, mercado de verduras, verduraria, verdureiro, verdurão, sacolão, centro de abastecimento, Ceasa, barraca de frutas e cereais em shopping center, banca de legumes, feira orgânica, feira, feira livre, feira livre de alimentos, feira do agricultor
Padarias e confeitarias	Padaria, depósito de pães, posto de pão, panificadora, casa de bolo, loja de bolo, confeitaria, doceria, loja de biscoito, confeitaria
Açougues	Açougue, casa de carnes, abatedouro, mercado de carnes, frigorifico em geral, butique de carnes, frigos, carniceria
Ambulantes de alimentos	Vendedor ambulante, ambulante, barraca (vendedor ambulante, treiller ou quiosque), carrinho de alimentos, carrocinha de alimentos, carrocinha, pit dog, carrinho de pipoca, leiteiro ambulante, padeiro ambulante, peixaria ambulante, food truck, regatão (vendedor ambulante embarcado)
Lanchonetes	Lanchonete, sorveteria, pizzaria, lancheria, pastelaria, cafeteria, lanchonete popular, loja de salgadinhos (lanche), pamonharia, café e bar, esfirraria, casa da esfira, creperia, vitaminosa
Bares	Taberna, boteco, botiquim (bar), bar, barzinho, choperia, baiuca, bolicho, tendinha (bar), uisqueria, pub, cachaçaria, copo sujo, scoth bar, adega
Restaurantes	Restaurante, churrascaria, bar e restaurante, marmitaria (serve refeições), restaurante de comidas típicas, *buffet*, peixaria (restaurante), self-service, restaurante popular, kilão, casa de massas, casa de comidas típicas, casa de assados, galeteria, frangueria (galeteria), vendedor de frango assado
Lojas de conveniência	Posto de combustível (loja de conveniência), loja de conveniência, posto de combustível, posto de gasolina
Peixarias	Peixaria, mercado de peixes, feira de peixes, balança do peixe
Bombonieres	Doceria, bomboniere, loja de balas, casa de doces, fábrica de bombom, deposito de doces e balas, loja de balas, doces e bombons
Varejistas de laticínios e frios	Loja de frios, loja de laticínios, laticínio, queijaria, vacaria (laticínios), distribuidora de leite, casa de queijo, leiteria
Cantinas	Escola, colégio, cantina, associação (recreação, futebol, clube, etc.), estabelecimento escolar, universidade, faculdade, cursinho, hospital público federal, hospital público estadual, hospital, público municipal, clube esportivo, instituto de ensino superior, creche, posto de saúde público federal, posto de saúde público estadual, posto de saúde público municipal, orgãos públicos, curso supletivo, curso profissionalizante, grupo escolar, escola de informática, curso pré-vestibular, escola de artes marciais, escola de dança, escola maternal, curso profissionalizante, escola agrotécnica, escola de música, escola de esportes, cursos preparatórios, escola de artes, escola de artesanato, escola de idiomas, hospital não especificado, posto de saúde público não especificado, estabelecimento de ensino, estabelecimento escolar, cursinho de matérias isoladas, escola isolada, auto-escola, curso supletivo
Vendas de alimentos congelados e prontos para consumo	Rotisseria, marmitex, delivery de comida chinesa, entrega em domicílio (venda por telefone), delicatesse, loja de alimentos congelados, loja de produtos congelados, loja de pão de queijo congelado, loja de comidas congeladas
Outros	Farmácia, drogaria, loja de departamento, banca de jornais, igreja, quermesse, bazar, loja de importados, telemarketing, loja de cesta de café da manhã, internet, deposito em geral, fabricante, fábrica, posto de fábrica, home center

Tabela suplementar 1. Percentual médio (%) e intervalo de confiança (IC95%) da contribuição dos locais de aquisição em relação à disponibilidade domiciliar de gramas de alimentos no Brasil e nas Grandes Regiões. Brasil, 2017-2018. Brasil, 2017-2018 (n = 57.920 domicílios)

Locais de aquisição de alimentos	Percentual (%) médio de gramas					
	Brasil	Norte	Nordeste	Sudeste	Sul	Centro-Oeste
	% (IC95%)	% (IC95%)	% (IC95%)	% (IC95%)	% (IC95%)	% (IC95%)
Supermercados	67,97 (65,33;70,60)	52,79 (46,76;58,82)	48,58 (46,63;50,54)	73,70 (68,89;78,51)	84,63 (79,02;90,21)	81,53 (79,56;83,48)
Minimercados e mercearias	9,85 (8,76;10,95)	19,56 (15,21;23,87)	21,42 (19,53;23,26)	5,01 (3,99;6,02)	2,92 (2,26;3,55)	3,33 (2,35;4,29)
Hortifrutigranjeiros	6,94 (6,30;7,57)	6,44 (4,87;7,98)	11,33 (9,91;12,70)	6,32 (5,33;7,29)	2,76 (2,11;3,39)	4,64 (3,69;5,57)
Padarias e confeitarias	5,76 (5,31;6,19)	5,82 (4,52;7,12)	7,08 (6,51;7,63)	6,33 (5,49;7,15)	2,55 (2,12;2,96)	4,49 (3,48;5,50)
Açougues	2,77 (2,52;3,00)	5,22 (4,36;6,07)	4,18 (3,73;4,64)	2,24 (1,88;2,57)	0,94 (0,70;1,16)	2,38 (1,94;2,80)
Ambulantes de alimentos	2,25 (1,86;2,61)	3,56 (2,19;4,91)	4,82 (3,77;5,84)	1,08 (0,78;1,38)	1,12 (0,81;1,41)	1,22 (0,95;1,46)
Lanchonetes	0,62 (0,49;0,72)	1,10 (0,12;2,08)	0,33 (0,26;0,39)	0,77 (0,61;0,93)	0,41 (0,27;0,54)	0,59 (0,43;0,71)
Restaurantes	0,36 (0,30;0,40)	0,47 (0,34;0,59)	0,39 (0,29;0,49)	0,32 (0,23;0,40)	0,26 (0,17;0,34)	0,49 (0,28;0,70)
Vendas de alimentos congelados e prontos para consumo	0,34 (0,23;0,42)	0,84 (0,13;1,53)	0,45 (0,27;0,64)	0,27 (0,13;0,40)	0,19 (0,08;0,30)	0,03 (0,01;0,05)
Bares	0,24 (0,19;0,29)	1,12 (0,64;1,60)	0,17 (0,08;0,26)	0,18 (0,13;0,22)	0,17 (0,06;0,27)	0,17 (0,07;0,28)
Lojas de conveniência	0,15 (0,08;0,21)	0,19 (0,01;0,38)	0,18 (0,04;0,32)	0,14 (0,02;0,24)	0,06 (0,01;0,11)	0,16 (0,06;0,27)
Peixarias	0,11 (0,08;0,13)	0,43 (0,28;0,57)	0,14 (0,10;0,19)	0,06 (0,03;0,09)	0,04 (0,01;0,07)	0,07 (0,02;0,13)
Bombonieres	0,06 (0,04;0,09)	0,01 (0,00;0,02)	0,03 (0,00;0,07)	0,10 (0,05;0,16)	0,06 (0,01;0,12)	0,02 (0,00;0,04)
Varejistas de laticínios e frios	0,05 (0,03;0,07)	0,02 (-0,02;0,07)	0,07 (0,02;0,11)	0,05 (0,02;0,07)	0,06 (0,02;0,11)	0,01 (0,00;0,02)
Cantinas	0,02 (0,00;0,04)	0,01 (-0,00;0,03)	0,02 (-0,00;0,04)	0,00 (0,00;0,01)	0,03 (-0,01;0,09)	0,11 (-0,09;0,32)
Outros	2,51 (-0,25;5,29)	2,42 (0,23;4,60)	0,81 (0,52;1,09)	3,43 (-2,49;9,31)	3,80 (-2,46;10,06)	0,76 (0,19;1,34)
**Total**	**100,00**	**100,00**	**100,00**	**100,00**	**100,00**	**100,00**

Tabela suplementar 2. Percentual médio (%) e intervalo de confiança (IC95%) da contribuição de cada grupo de alimentos da Nova para o total de gramas adquiridas dentro de cada local de aquisição e sua classificação conforme o sistema de classificação Locais-Nova. Brasil, 2017-2018 (n = 57.920 domicílios)

Locais de aquisição de alimentos	Percentual (%) médio de gramas		
	G1+G2[a]	G3[b]	G4[c]
	% (IC95%)	% (IC95%)	% (IC95%)
Critérios para classificação dos locais fontes de aquisição de alimentos - Brasil	64,65 (63,51;65,81)	8,87 (8,57;9,17)	26,48 (25,38;27,56)
Supermercados	69,29 (68,27;70,31)	3,67 (3,48;3,76)	27,04 (26,02;28,03)
Minimercados, Mercearias	64,49 (62,83;66,16)	4,93 (4,58;5,25)	30,58 (28,95;32,20)
Hortifrutigranjeiros	93,69 (92,48;94,92)	1,81 (1,42;2,15)	4,50 (3,39;5,60)
Padaria e Confeitaria	18,28 (16,71;19,85)	54,90 (53,16;56,65)	26,82 (25,25;28,39)
Açougues	87,75 (86,48;89,04)	2,65 (2,11;3,14)	9,60 (8,38;10,82)
Ambulantes de Alimentos	74,76 (71,50;78,04)	8,72 (6,95;10,48)	16,52 (13,51;19,48)
Lanchonetes	11,57 (8,44;14,72)	0,89 (0,41;1,38)	87,54 (84,33;90,70)
Restaurantes	84,08 (80,57;87,56)	0,81 (0,09;1,54)	15,11 (11,64;18,58)
Vendas de alimentos congelados e prontos para consumo	43,23 (33,43;53,01)	2,77 (1,11;4,42)	54,00 (44,15;63,84)
Bares	26,17 (20,03;32,33)	9,37 (5,23;13,51)	64,46 (57,54;71,33)
Lojas de Conveniência	7,32 (2,35;12,17)	13,63 (4,22;23,05)	79,05 (69,18;88,92)
Peixaria	99,65 (98,94;100,43)	0,35 (-0,34;1,05)	0,00 (0,00;0,00)
Bomboniere	9,54 (3,02;16,06)	3,80 (1,08;6,53)	86,66 (79,76;93,51)
Varejista de Laticínios e Frios	32,12 (20,12;44,13)	49,05 (37,91;60,14)	18,83 (10,15;27,51)
Cantinas	44,96 (21,89;68,04)	11,62 (-6,96;30;17)	43,42 (18,99;67,85)
Outros	46,74 (40,35;53,13)	3,53 (1,92;5,11)	49,73 (43,73;56,14)

Legenda:
(verde) Local fonte de aquisição de alimentos *in natura* ou minimamente processados e ingredientes culinários
(amarelo) Local fonte de aquisição de alimentos processados
(vermelho) Local fonte de aquisição de alimentos ultraprocessados

Nota: [a]G1+G2=*in natura* ou minimamente processados (G1) e ingredientes culinários (G2); [b]G3=alimentos processados; [c]G4=alimentos ultraprocessados.

Tabela suplementar 3. Percentual médio (%) e intervalo de confiança (IC95%) dos alimentos adquiridos dentro de cada local de aquisição segunda a classificação Nova em relação à disponibilidade domiciliar na região Norte do Brasil. Brasil, 2017-2018 (n = 57.920 domicílios)

Locais de aquisição de alimentos	Percentual (%) médio de gramas		
	G1+G2[a]	G3[b]	G4[c]
	% (IC95%)	% (IC95%)	% (IC95%)
Critérios para classificação dos locais fontes de aquisição de alimentos - Norte	71,63 (68,89;74,38)	7,60 (6,41;8,81)	20,77 (18,38;23,17)
Supermercados	75,73 (73,48;77,99)	3,18 (2,61;3,71)	21,09 (18,75;23,32)
Minimercados e mercearias	71,53 (68,18;74;88)	3,55 (2,99;4,09)	24,92 (21,76;28,06)
Hortifrutigranjeiros	95,81 (93,72;97,90)	1,24 (0,76;1,70)	2,95 (0,82;5,06)
Padarias e confeitarias	11,39 (8,62;14,15)	66,76 (61,95;71,55)	21,85 (18,84;24,85)
Açougues	95,87 (94,44;97,31)	1,53 (0,69,2,33)	2,60 (1,54;3,67)
Ambulantes de alimentos	80,86 (73,26;88,45)	3,65 (1,92;5,36)	15,49 (8,33;22,66)
Lanchonetes	35,38 (19,67;51,08)	0,59 (-0,20;1,38)	64,03 (48,34;79,71)
Restaurantes	90,19 (83,53;96,83)	0,71 (-0,57;1,99)	9,10 (2,57;15,63)
Vendas de alimentos congelados e prontos para consumo	46,01 (4,89;87,12)	0,00 (0,00;0,00)	53,99 (12,87;95,10)
Bares	52,06 (40,24;63,88)	11,76 (3,41;20,09)	36,18 (24,56;47,80)
Lojas de conveniência	0,00 (0,00;0,00)	0,17 (-0,09;0,42)	99,83 (99,57;100,09)
Peixarias	100 (100;100)	0,00 (0,00;0,00)	0,00 (0,00;0,00)
Bombonieres	0,00 (0,00;0,00)	4,19 (-0,83;9,22)	95,81 (90,77;100,83)
Varejistas de laticínios e frios	34,85 (-20,75;90,46)	31,66 (-21,32;84,63)	33,49 (-21,07;88,06)
Cantinas	56,02 (-2,74;114,79)	0,00 (0,00;0,00)	43,98 (-14,79;102,74)
Outros	49,07 (30,86;67,28)	1,68 (-0,58;3,95)	49,25 (31,14;67,33)

Legenda:

(verde) Local fonte de aquisição de alimentos *in natura* ou minimamente processados e ingredientes culinários

(amarelo) Local fonte de aquisição de alimentos processados

(rosa) Local fonte de aquisição de alimentos ultraprocessados

Nota: [a]G1+G2=*in natura* ou minimamente processados (G1) e ingredientes culinários (G2); [b]G3=alimentos processados; [c]G4=alimentos ultraprocessados.

Tabela suplementar 4. Percentual médio (%) e intervalo de confiança (IC95%) dos alimentos adquiridos dentro de cada local de aquisição segunda a classificação Nova em relação à disponibilidade domiciliar na região Nordeste do Brasil. Brasil, 2017-2018 (n = 57.920 domicílios)

Locais de aquisição de alimentos	Percentual (%) médio de gramas		
	G1+G2[a]	G3[b]	G4[c]
	% (IC95%)	% (IC95%)	% (IC95%)
Critérios para classificação dos locais fontes de aquisição de alimentos - Nordeste	64,22 (61,21;67,23)	8,21 (7,60;8,80)	27,57 (24,68;30,46)
Supermercados	72,70 (70,93;74,46)	3,58 (3,25;3,90)	23,72 (22,06;25,38)
Minimercados e mercearias	63,22 (60,05;66,38)	4,42 (4,93;4,90)	32,36 (29,26;35,45)
Hortifrutigranjeiros	96,23 (95,41;97,04)	1,59 (1,27;1,90)	2,18 (1,40;2,94)
Padarias e confeitarias	14,49 (12,89;16,07)	67,20 (64,94;69,45)	18,31 (16,31;20,31)
Açougues	92,58 (91,42;93,74)	3,94 (2,95;4,89)	3,48 (2,75;4,21)
Ambulantes de alimentos	75,51 (70,38;80,63)	9,29 (7,08;11,48)	15,20 (9,97;20,42)
Lanchonetes	11,86 (7,72;15,98)	2,09 (0,45;3,74)	86,05 (81,30;90,78)
Restaurantes	86,11 (81,29;90,89)	0,99 (-0,73;2,73)	12,90 (8,54;17,26)
Vendas de alimentos congelados e prontos para consumo	29,50 (17,41;41,59)	7,27 (2,68;11,84)	63,23 (50,11;76,34)
Bares	30,49 (19,07;41,90)	4,63 (1,13;8,11)	64,88 (53,28;76,48)
Lojas de conveniência	13,32 (-0,27;26,92)	7,05 (-3,88;17,98)	79,63 (62,97;96,27)
Peixarias	98,88 (96,73;101,04)	1,12 (-1,04;3,26)	0,00 (0,00;0,00)
Bombonieres	16,65 (2,38;30,92)	6,86 (-3,01;16,73)	76,49 (60,60;92,37)
Varejistas de laticínios e frios	49,32 (31,53,67,12)	38,15 (22,48;53,80)	12,53 (5,54;19,50)
Cantinas	58,65 (27,22;90,09)	8,38 (-1,38;18,12)	32,97 (3,53;62,39)
Outros	57,42 (47,67;67,17)	3,51 (0,56;6,43)	39,07 (29,51;48,62)

Legenda:
- Local fonte de aquisição de alimentos *in natura* ou minimamente processados e ingredientes culinários
- Local fonte de aquisição de alimentos processados
- Local fonte de aquisição de alimentos ultraprocessados

Nota: [a]G1+G2=*in natura* ou minimamente processados (G1) e ingredientes culinários (G2); [b]G3=alimentos processados; [c]G4=alimentos ultraprocessados.

Tabela suplementar 5. Percentual médio (%) e intervalo de confiança (IC95%) dos alimentos adquiridos dentro de cada local de aquisição segunda a classificação Nova em relação à disponibilidade domiciliar na região Sudeste do Brasil. Brasil, 2017-2018 (n = 57.920 domicílios)

Locais de aquisição de alimentos	Percentual (%) médio de gramas		
	G1+G2[a]	G3[b]	G4[c]
	% (IC95%)	% (IC95%)	% (IC95%)
Critérios para classificação dos locais fontes de aquisição de alimentos - Sudeste	63,07 (61,57;64,60)	9,41 (8,93;9,89)	27,52 (26,03;29,00)
Supermercados	67,77 (66,08;69,45)	3,75 (3,52;3,96)	28,48 (26,91;30,06)
Minimercados e mercearias	62,30 (59,37;65,25)	5,47 (4,84;6,08)	32,23 (29,28;35,16)
Hortifrutigranjeiros	93,23 (91,04;95,42)	1,29 (0,95;1,62)	5,48 (3,42;7,52)
Padarias e confeitarias	23,63 (20,63;26,60)	50,43 (47,78;53,06)	25,94 (23,26;28,63)
Açougues	84,09 (81,83;86,35)	2,44 (1,57;3,30)	13,47 (11,31;15,62)
Ambulantes de alimentos	69,96 (63,86;76,07)	10,69 (7,21;14,15)	19,35 (13,78;24,91)
Lanchonetes	9,72 (4,43;15,00)	0,43 (0,10;0,76)	89,85 (84,59;95,08)
Restaurantes	81,07 (74,67;87,47)	0,96 (-0,29;2,18)	17,97 (11,54;24,41)
Vendas de alimentos congelados e prontos para consumo	51,86 (34,45;69,27)	1,95 (-0,34;4,22)	46,19 (28,67;63,71)
Bares	24,10 (13,93;34,26)	12,03 (4,62;19,43)	63,87 (52,43;75,31)
Lojas de conveniência	1,28 (-0,59;3,15)	21,39 (3,45;39,33)	77,33 (59,81;94,83)
Peixarias	100 (100;100)	0,00 (0,00;0,00)	0,00 (0,00;0,00)
Bombonieres	7,42 (0,01;14,79)	4,05 (0,66;7,44)	88,53 (80,61;96,46)
Varejistas de laticínios e frios	21,02 (3,24;38,81)	54,46 (36,69;72,21)	24,52 (8,73;40,29)
Cantinas	22,87 (-8,37;54,11)	20,66 (16,68;58,00)	56,47 (13,48;99,45)
Outros	36,78 (25,85;47,68)	2,59 (0,27;4,92)	60,63 (49,72;71,53)

Legenda:

(verde) Local fonte de aquisição de alimentos *in natura* ou minimamente processados e ingredientes culinários

(amarelo) Local fonte de aquisição de alimentos processados

(vermelho) Local fonte de aquisição de alimentos ultraprocessados

Nota: [a]G1+G2=*in natura* ou minimamente processados (G1) e ingredientes culinários (G2); [b]G3=alimentos processados; [c]G4=alimentos ultraprocessados.

Tabela suplementar 6. Percentual médio (%) e intervalo de confiança (IC95%) dos alimentos adquiridos dentro de cada local de aquisição segunda a classificação Nova em relação à disponibilidade domiciliar na região Sul do Brasil. Brasil, 2017-2018 (n = 57.920 domicílios)

Locais de aquisição de alimentos	Percentual (%) médio de gramas		
	G1+G2[a]	G3[b]	G4[c]
	% (IC95%)	% (IC95%)	% (IC95%)
Critérios para classificação dos locais fontes de aquisição de alimentos - Sul	64,21 (62,80;65,62)	9,21 (8,75;9,67)	26,58 (25,35;27,81)
Supermercados	67,12 (65,77;68,45)	4,31 (4,06;4,55)	28,57 (27,32;29,81)
Minimercados e mercearias	64,17 (62,45;65,88)	4,70 (4,06;5,32)	31,13 (29,62;32,64)
Hortifrutigranjeiros	89,94 (85,54;94,33)	3,72 (1,62;5,80)	6,34 (2,83;6,34)
Padarias e confeitarias	18,18 (15,41;20,95)	44,29 (40,99;47,59)	37,53 (33,93;41,10)
Açougues	85,09 (81,22;88,96)	1,43 (0,23;2,60)	13,48 (9,92;17,03)
Ambulantes de alimentos	80,96 (75,57;86,36)	4,42 (2,43;6,39)	14,62 (9,40;19,82)
Lanchonetes	8,79 (2,04;15,56)	0,02 (-0,01;0,04)	91,19 (84,42;97,94)
Restaurantes	89,96 (84,26;95,66)	0,29 (-0,27;0,84)	9,75 (4,04;15,44)
Vendas de alimentos congelados e prontos para consumo	33,45 (20,21;46,70)	0,17 (-0,15;0,48)	66,38 (52,98;79,75)
Bares	14,29 (2,36;26,22)	8,75 (-0,79;18,28)	76,96 (61,08;92,84)
Lojas de conveniência	17,23 (-1,53;36,01)	6,06 (-1,14;13,20)	76,71 (57,97;95,43)
Peixarias	100 (100;100)	0,00 (0,00;0,00)	0,00 (0,00;0,00)
Bombonieres	16,51 (-10,23;43,25)	0,00 (0,00;0,00)	83,49 (80,61;96,46)
Varejistas de laticínios e frios	38,59 (3,23;73,94)	60,19 (24,97;95,42)	1,22 (-0,42;2,85)
Cantinas	86,24 (58,25;114,22)	0,00 (0,00;0,00)	13,76 (-14,22;41,74)
Outros	53,90 (41,62;66,20)	7,84 (2,12;13,54)	38,26 (26,42;50,07)

Legenda:

(verde) Local fonte de aquisição de alimentos *in natura* ou minimamente processados e ingredientes culinários

(amarelo) Local fonte de aquisição de alimentos processados

(vermelho) Local fonte de aquisição de alimentos ultraprocessados

Nota: [a]G1+G2=*in natura* ou minimamente processados (G1) e ingredientes culinários (G2); [b]G3=alimentos processados; [c]G4=alimentos ultraprocessados.

Tabela suplementar 7. Percentual médio (%) e intervalo de confiança (IC95%) dos alimentos adquiridos dentro de cada local de aquisição segunda a classificação Nova em relação à disponibilidade domiciliar na região Centro-Oeste do Brasil. Brasil, 2017-2018 (n = 57.920 domicílios)

Locais de aquisição de alimentos	Percentual (%) médio de gramas		
	G1+G2[a]	G3[b]	G4[c]
	% (IC95%)	% (IC95%)	% (IC95%)
Critérios para classificação dos locais fontes de aquisição de alimentos - Centro-Oeste	69,37 (66,96;71,77)	8,58 (7,88;9,29)	22,05 (19,92;24,17)
Supermercados	73,96 (72,76;75,16)	3,15 (2,77;3,53)	22,89 (20,86;24,92)
Minimercados e mercearias	69,30 (65,59;73,01)	4,88 (3,88;5,82)	25,82 (22,25;29,21)
Hortifrutigranjeiros	93,10 (90,22;95,96)	2,11 (1,25;2,96)	4,79 (2,16;7,42)
Padarias e confeitarias	20,44 (16,36;24,53)	49,29 (45,35;53,21)	30,27 (30,26;33,78)
Açougues	89,24 (87,26;91;22)	2,57 (1,27;3,84)	8,19 (6,42;9,95)
Ambulantes de alimentos	81,84 (74,97;88,72)	8,77 (2,62;14,90)	9,39 (4,47;14,29)
Lanchonetes	6,08 (3,08;9,09)	1,83 (-0,80;4,44)	92,09 (88,15;96,02)
Restaurantes	77,54 (66,96;88,09)	0,59 (-0,20;1,28)	21,87 (11,46;32,29)
Vendas de alimentos congelados e prontos para consumo	52,76 (25,92;79,62)	0,00 (0,00;0,00)	47,24 (20,37;74,07)
Bares	10,90 (0,08;21,90)	2,19 (-2,00;6,37)	86,91 (75,70;98,10)
Lojas de conveniência	11,17 (0,69;21,62)	10,82 (-4,95;26,61)	78,01 (59,84;96,18)
Peixarias	100 (100;100)	0,00 (0,00;0,00)	0,00 (0,00;0,00)
Bombonieres	0,00 (0,00;0,00)	0,00 (0,00;0,00)	100 (100;100)
Varejistas de laticínios e frios	8,52 (-0,25;17,31)	45,06 (27,79;62,32)	46,42 (27,34;65,47)
Cantinas	52,17 (4,62;99,72)	0,40 (-0,39;1,19)	47,43 (-0,21;95,16)
Outros	53,82 (39,29;68,36)	1,65 (-0,16;3,46)	44,53 (29,90;59,13)

Legenda:
Local fonte de aquisição de alimentos *in natura* ou minimamente processados e ingredientes culinários
Local fonte de aquisição de alimentos processados
Local fonte de aquisição de alimentos ultraprocessados

Nota: [a]G1+G2=*in natura* ou minimamente processados (G1) e ingredientes culinários (G2); [b]G3=alimentos processados; [c]G4=alimentos ultraprocessadoss

Supplementary Box 1. Food purchasing place grouping. Brazil, 2017-2018

**Grupos**	**Agrupamento detalhado**
Supermarkets	Hypermarket, wholesale hypermarket, wholesaler, wholesale store, supermarket, wholesale supermarket, commercial
Small markets and grocery stores	Grocery store, market (store), small market (grocery store), bodega (grocery store), bodega (store), general store, mini-market (store), municipal market, state market, small market, emporium, birosca, retail warehouse, dry and wet goods (store), public market, granary, sales point, warehouse, booth (grocery store), minibox, flour house, casa do norte, wholesale warehouse, wholesale granary
Fruits, vegetables, and farm products	Greengrocer, fruit store, fruit stand, fruit market, greengrocer, vegetable market, vegetable stand, vegetable store, fruit and vegetable store, food supply center, Ceasa, fruit and cereal stall in a shopping center, organic food market, market, street market, food market, farmer's market
Bakeries and confectionaries	Bakery, bread store, bread station, bakery, cake house, cake shop, confectionery, sweet shop, biscuit shop
Butcher shops	Butcher's shop, meat shop, slaughterhouse, meat market, slaughterhouse in general, meat store, cold stores, butchery
Food street vendors	Street vendor, hawker, stall (hawker, trailer or kiosk), food cart, cart, pit dog, popcorn cart, street milkman, street baker, street fishmonger, food truck, regatão (on-board street vendor)
Snack bars	Snack bar, ice cream parlor, pizzeria, pastry shop, cafeteria, people's snack bar, snack shop, pamonharia, cafe and bar, sfiha store, sfiha house, creperie, milk shake bar
Bars	Tavern, bar, botiquim (bar), bar, taproom, baiuca, bolicho, stall (bar), whiskey shop, pub, cachaçaria, copo sujo, Scotch bar, wine cellar
Restaurants	Restaurant, steakhouse, bar and restaurant, marmitaria (serves meals), typical food restaurant, buffet, fishmonger (restaurant), self-service, people's restaurant, kilão, pasta house, typical food house, roast house, chicken spit, chicken restaurant (checkin spit), roast chicken vendor
Convenience stores	Gas station (convenience store), convenience store, fuel station, gas station
Fish markets	Fishmonger, indoor fish market, outdoor fish market, fish scales
Candy stores	Sweet shop, candy store, candy shop, candy house, candy factory, candy warehouse, candy, sweets and bonbons store
Dairy and cold cuts retailers	Delicatessen, dairy store, dairy, cheese shop, dairy products (dairy), milk distributor, cheese house, milk outlet
Canteens	School, college, canteen, association (recreation, football, club, etc.), school establishment, university, college, cram school, federal public hospital, state public hospital, municipal public hospital, sports club, higher education institute, daycare center, federal public health center, state public health center, municipal public health center, public agencies, supplementary course, vocational course, school group, IT school, pre-university course, martial arts school, dance school, nursery school, vocational course, agrotechnology school, music school, sports school, preparatory courses, art school, craft school, language school, unspecified hospital, unspecified public health center, educational establishment, school establishment, single subjects course, single class school, driving school, supplementary course
Frozen and ready-to-eat food vendors	Rotisserie, lunchbox, Chinese food delivery, home delivery (sale by telephone), delicatessen, frozen food store, frozen product store, frozen cheese bread store, frozen food store
Others	Pharmacy, drugstore, department store, newsstand, church, fair, bazaar, import store, telemarketing, breakfast basket store, internet, general store, manufacturer, factory, factory station, home store

Tabela suplementar 1. Average percentages (%) and confidence intervals (95%CI) of the share of purchasing places in relation to household availability of grams of food. Brazil and Brazilian Regions, 2017-2018 (n=57,920)

Food purchasing places	Average percentage (%) grams					
	Brazil	North	Northeast	Southeast	South	Midwest
	% (IC95%)	% (IC95%)	% (IC95%)	% (IC95%)	% (IC95%)	% (IC95%)
Supermarkets	67.97 (65.33;70.60)	52.79 (46.76;58.82)	48.58 (46.63;50.54)	73.70 (68.89;78.51)	84.63 (79.02;90.21)	81.53 (79.56;83.48)
Small markets and grocery stores	9.85 (8.76;10.95)	19.56 (15.21;23.87)	21.42 (19.53;23.26)	5.01 (3.99;6.02)	2.92 (2.26;3.55)	3.33 (2.35;4.29)
Fruits. vegetables. and farm products	6.94 (6.30;7.57)	6.44 (4.87;7.98)	11.33 (9.91;12.70)	6.32 (5.33;7.29)	2.76 (2.11;3.39)	4.64 (3.69;5.57)
Bakeries and confectionaries	5.76 (5.31;6.19)	5.82 (4.52;7.12)	7.08 (6.51;7.63)	6.33 (5.49;7.15)	2.55 (2.12;2.96)	4.49 (3.48;5.50)
Butcher shops	2.77 (2.52;3.00)	5.22 (4.36;6.07)	4.18 (3.73;4.64)	2.24 (1.88;2.57)	0.94 (0.70;1.16)	2.38 (1.94;2.80)
Food street vendors	2.25 (1.86;2.61)	3.56 (2.19;4.91)	4.82 (3.77;5.84)	1.08 (0.78;1.38)	1.12 (0.81;1.41)	1.22 (0.95;1.46)
Snack bars	0.62 (0.49;0.72)	1.10 (0.12;2.08)	0.33 (0.26;0.39)	0.77 (0.61;0.93)	0.41 (0.27;0.54)	0.59 (0.43;0.71)
Restaurants	0.36 (0.30;0.40)	0.47 (0.34;0.59)	0.39 (0.29;0.49)	0.32 (0.23;0.40)	0.26 (0.17;0.34)	0.49 (0.28;0.70)
Frozen and ready-to-eat food vendors	0.34 (0.23;0.42)	0.84 (0.13;1.53)	0.45 (0.27;0.64)	0.27 (0.13;0.40)	0.19 (0.08;0.30)	0.03 (0.01;0.05)
Bars	0.24 (0.19;0.29)	1.12 (0.64;1.60)	0.17 (0.08;0.26)	0.18 (0.13;0.22)	0.17 (0.06;0.27)	0.17 (0.07;0.28)
Convenience stores	0.15 (0.08;0.21)	0.19 (0.01;0.38)	0.18 (0.04;0.32)	0.14 (0.02;0.24)	0.06 (0.01;0.11)	0.16 (0.06;0.27)
Fish markets	0.11 (0.08;0.13)	0.43 (0.28;0.57)	0.14 (0.10;0.19)	0.06 (0.03;0.09)	0.04 (0.01;0.07)	0.07 (0.02;0.13)
Candy stores	0.06 (0.04;0.09)	0.01 (0.00;0.02)	0.03 (0.00;0.07)	0.10 (0.05;0.16)	0.06 (0.01;0.12)	0.02 (0.00;0.04)
Dairy and cold cuts retailers	0.05 (0.03;0.07)	0.02 (-0.02;0.07)	0.07 (0.02;0.11)	0.05 (0.02;0.07)	0.06 (0.02;0.11)	0.01 (0.00;0.02)
Canteens	0.02 (0.00;0.04)	0.01 (-0.00;0.03)	0.02 (-0.00;0.04)	0.00 (0.00;0.01)	0.03 (-0.01;0.09)	0.11 (-0.09;0.32)
Others	2.51 (-0.25;5.29)	2.42 (0.23;4.60)	0.81 (0.52;1.09)	3.43 (-2.49;9.31)	3.80 (-2.46;10.06)	0.76 (0.19;1.34)
**Total**	**100.00**	**100.00**	**100.00**	**100.00**	**100.00**	**100.00**

Supplementary Table 2. Average percentages (%) and confidence intervals (95%CI) of the share of each Nova Classification food group in total grams acquired at each purchasing place. Brazil, 2017-2018 (n = 57.920)

Food purchasing places	Average percentage (%) grams		
	G1+G2[a]	G3[b]	G4[c]
	% (95%CI)	% (95%CI)	% (95%CI)
Criteria for classifying food source purchasing places - Brazil	64.65 (63.51;65.81)	8.87 (8.57;9.17)	26.48 (25.38;27.56)
Supermarkets	69.29 (68.27;70.31)	3.67 (3.48;3.76)	27.04 (26.02;28.03)
Small markets and grocery stores	64.49 (62.83;66.16)	4.93 (4.58;5.25)	30.58 (28.95;32.20)
Fruits. vegetables. and farm products	93.69 (92.48;94.92)	1.81 (1.42;2.15)	4.50 (3.39;5.60)
Bakeries and confectionaries	18.28 (16.71;19.85)	54.90 (53.16;56.65)	26.82 (25.25;28.39)
Butcher shops	87.75 (86.48;89.04)	2.65 (2.11;3.14)	9.60 (8.38;10.82)
Food street vendors	74.76 (71.50;78.04)	8.72 (6.95;10.48)	16.52 (13.51;19.48)
Snack bars	11.57 (8.44;14.72)	0.89 (0.41;1.38)	87.54 (84.33;90.70)
Restaurants	84.08 (80.57;87.56)	0.81 (0.09;1.54)	15.11 (11.64;18.58)
Frozen and ready-to-eat food vendors	43.23 (33.43;53.01)	2.77 (1.11;4.42)	54.00 (44.15;63.84)
Bars	26.17 (20.03;32.33)	9.37 (5.23;13.51)	64.46 (57.54;71.33)
Convenience stores	7.32 (2.35;12.17)	13.63 (4.22;23.05)	79.05 (69.18;88.92)
Fish markets	99.65 (98.94;100.43)	0.35 (-0.34;1.05)	0.00 (0.00;0.00)
Candy stores	9.54 (3.02;16.06)	3.80 (1.08;6.53)	86.66 (79.76;93.51)
Dairy and cold cuts retailers	32.12 (20.12;44.13)	49.05 (37.91;60.14)	18.83 (10.15;27.51)
Canteens	44.96 (21.89;68.04)	11.62 (-6.96;30;17)	43.42 (18.99;67.85)
Others	46.74 (40.35;53.13)	3.53 (1.92;5.11)	49.73 (43.73;56.14)

Legend:

(green) Source of acquisition of unprocessed or minimally processed foods and processed culinary ingredients

(yellow) Source of acquisition of processed foods

(red) Source of acquisition of ultra-processed foods

Note: [a]G1+G2=unprocessed or minimally processed food (G1) and processed culinary ingredients (G2); [b]G3=processed foods; [c]G4=ultra-processed foods.

Supplementary Table 3. Average percentages (%) and confidence intervals (95%CI) of the share of each Nova Classification food group in total grams acquired at each purchasing place. Northern Region, Brazil, 2017-2018 (n = 57,920)

Food purchasing places	Average percentage (%) grams		
	G1+G2[a]	G3[b]	G4[c]
	% (95%CI)	% (95%CI)	% (95%CI)
Criteria for classifying food source purchasing places - North	71.63 (68.89;74.38)	7.60 (6.41;8.81)	20.77 (18.38;23.17)
Supermarkets	75.73 (73.48;77.99)	3.18 (2.61;3.71)	21.09 (18.75;23.32)
Small markets and grocery stores	71.53 (68.18;74;88)	3.55 (2.99;4.09)	24.92 (21.76;28.06)
Fruits, vegetables, and farm products	95.81 (93.72;97.90)	1.24 (0.76;1.70)	2.95 (0.82;5.06)
Bakeries and confectionaries	11.39 (8.62;14.15)	66.76 (61.95;71.55)	21.85 (18.84;24.85)
Butcher shops	95.87 (94.44;97.31)	1.53 (0.69.2.33)	2.60 (1.54;3.67)
Food street vendors	80.86 (73.26;88.45)	3.65 (1.92;5.36)	15.49 (8.33;22.66)
Snack bars	35.38 (19.67;51.08)	0.59 (-0.20;1.38)	64.03 (48.34;79.71)
Restaurants	90.19 (83.53;96.83)	0.71 (-0.57;1.99)	9.10 (2.57;15.63)
Frozen and ready-to-eat food vendors	46.01 (4.89;87.12)	0.00 (0.00;0.00)	53.99 (12.87;95.10)
Bars	52.06 (40.24;63.88)	11.76 (3.41;20.09)	36.18 (24.56;47.80)
Convenience stores	0.00 (0.00;0.00)	0.17 (-0.09;0.42)	99.83 (99.57;100.09)
Fish markets	100.00 (100.00;100.00)	0.00 (0.00;0.00)	0.00 (0.00;0.00)
Candy stores	0.00 (0.00;0.00)	4.19 (-0.83;9.22)	95.81 (90.77;100.83)
Dairy and cold cuts retailers	34.85 (-20.75;90.46)	31.66 (-21.32;84.63)	33.49 (-21.07;88.06)
Canteens	56.02 (-2.74;114.79)	0.00 (0.00;0.00)	43.98 (-14.79;102.74)
Others	49.07 (30.86;67.28)	1.68 (-0.58;3.95)	49.25 (31.14;67.33)

Legend:

- (green) Source of acquisition of unprocessed or minimally processed foods and processed culinary ingredients
- (yellow) Source of acquisition of processed foods
- (red) Source of acquisition of ultra-processed foods

Note: [a]G1+G2=unprocessed or minimally processed food (G1) and processed culinary ingredients (G2); [b]G3=processed foods; [c]G4=ultra-processed foods.

Supplementary Table 4. Average percentages (%) and confidence intervals (95%CI) of the share of each Nova Classification food group in total grams acquired at each purchasing place. Northeast Region, Brazil, 2017-2018 (n = 57,920)

Food purchasing places	Average percentage (%) grams		
	G1+G2[a]	G3[b]	G4[c]
	% (95%CI)	% (95%CI)	% (95%CI)
Criteria for classifying food source purchasing places - Northeast	64.22 (61.21;67.23)	8.21 (7.60;8.80)	27.57 (24.68;30.46)
Supermarkets	72.70 (70.93;74.46)	3.58 (3.25;3.90)	23.72 (22.06;25.38)
Small markets and grocery stores	63.22 (60.05;66.38)	4.42 (4.93;4.90)	32.36 (29.26;35.45)
Fruits, vegetables, and farm products	96.23 (95.41;97.04)	1.59 (1.27;1.90)	2.18 (1.40;2.94)
Bakeries and confectionaries	14.49 (12.89;16.07)	67.20 (64.94;69.45)	18.31 (16.31;20.31)
Butcher shops	92.58 (91.42;93.74)	3.94 (2.95;4.89)	3.48 (2.75;4.21)
Food street vendors	75.51 (70.38;80.63)	9.29 (7.08;11.48)	15.20 (9.97;20.42)
Snack bars	11.86 (7.72;15.98)	2.09 (0.45;3.74)	86.05 (81.30;90.78)
Restaurants	86.11 (81.29;90.89)	0.99 (-0.73;2.73)	12.90 (8.54;17.26)
Frozen and ready-to-eat food vendors	29.50 (17.41;41.59)	7.27 (2.68;11.84)	63.23 (50.11;76.34)
Bars	30.49 (19.07;41.90)	4.63 (1.13;8.11)	64.88 (53.28;76.48)
Convenience stores	13.32 (-0.27;26.92)	7.05 (-3.88;17.98)	79.63 (62.97;96.27)
Fish markets	98.88 (96.73;101.04)	1.12 (-1.04;3.26)	0.00 (0.00;0.00)
Candy stores	16.65 (2.38;30.92)	6.86 (-3.01;16.73)	76.49 (60.60;92.37)
Dairy and cold cuts retailers	49.32 (31.53.67.12)	38.15 (22.48;53.80)	12.53 (5.54;19.50)
Canteens	58.65 (27.22;90.09)	8.38 (-1.38;18.12)	32.97 (3.53;62.39)
Others	57.42 (47.67;67.17)	3.51 (0.56;6.43)	39.07 (29.51;48.62)

Legend:
- (green) Source of acquisition of unprocessed or minimally processed foods and processed culinary ingredients
- (yellow) Source of acquisition of processed foods
- (red) Source of acquisition of ultra-processed foods

Note: [a]G1+G2=unprocessed or minimally processed food (G1) and processed culinary ingredients (G2); [b]G3=processed foods; [c]G4=ultra-processed foods.

Supplementary Table 5. Average percentages (%) and confidence intervals (95%CI) of the share of each Nova Classification food group in total grams acquired at each purchasing place. Southeast Region, Brazil, 2017-2018 (n = 57,920)

Food purchasing places	Average percentage (%) grams		
	G1+G2[a]	G3[b]	G4[c]
	% (95%CI)	% (95%CI)	% (95%CI)
Criteria for classifying food source purchasing places - Southeast	63.07 (61.57;64.60)	9.41 (8.93;9.89)	27.52 (26.03;29.00)
Supermarkets	67.77 (66.08;69.45)	3.75 (3.52;3.96)	28.48 (26.91;30.06)
Small markets and grocery stores	62.30 (59.37;65.25)	5.47 (4.84;6.08)	32.23 (29.28;35.16)
Fruits, vegetables, and farm products	93.23 (91.04;95.42)	1.29 (0.95;1.62)	5.48 (3.42;7.52)
Bakeries and confectionaries	23.63 (20.63;26.60)	50.43 (47.78;53.06)	25.94 (23.26;28.63)
Butcher shops	84.09 (81.83;86.35)	2.44 (1.57;3.30)	13.47 (11.31;15.62)
Food street vendors	69.96 (63.86;76.07)	10.69 (7.21;14.15)	19.35 (13.78;24.91)
Snack bars	9.72 (4.43;15.00)	0.43 (0.10;0.76)	89.85 (84.59;95.08)
Restaurants	81.07 (74.67;87.47)	0.96 (-0.29;2.18)	17.97 (11.54;24.41)
Frozen and ready-to-eat food vendors	51.86 (34.45;69.27)	1.95 (-0.34;4.22)	46.19 (28.67;63.71)
Bars	24.10 (13.93;34.26)	12.03 (4.62;19.43)	63.87 (52.43;75.31)
Convenience stores	1.28 (-0.59;3.15)	21.39 (3.45;39.33)	77.33 (59.81;94.83)
Fish markets	100.00 (100.00;100.00)	0.00 (0.00;0.00)	0.00 (0.00;0.00)
Candy stores	7.42 (0.01;14.79)	4.05 (0.66;7.44)	88.53 (80.61;96.46)
Dairy and cold cuts retailers	21.02 (3.24;38.81)	54.46 (36.69;72.21)	24.52 (8.73;40.29)
Canteens	22.87 (-8.37;54.11)	20.66 (16.68;58.00)	56.47 (13.48;99.45)
Others	36.78 (25.85;47.68)	2.59 (0.27;4.92)	60.63 (49.72;71.53)

Legend:
Source of acquisition of unprocessed or minimally processed foods and processed culinary ingredients
Source of acquisition of processed foods
Source of acquisition of ultra-processed foods

Note: [a]G1+G2=unprocessed or minimally processed food (G1) and processed culinary ingredients (G2); [b]G3=processed foods; [c]G4=ultra-processed foods.

Supplementary Table 6. Average percentages (%) and confidence intervals (95%CI) of the share of each Nova Classification food group in total grams acquired at each purchasing place. Southern Region, Brazil, 2017-2018 (n = 57,920)

Food purchasing places	Average percentage (%) grams		
	G1+G2[a]	G3[b]	G4[c]
	% (95%CI)	% (95%CI)	% (95%CI)
Criteria for classifying food source purchasing places - South	64.21 (62.80;65.62)	9.21 (8.75;9.67)	26.58 (25.35;27.81)
Supermarkets	67.12 (65.77;68.45)	4.31 (4.06;4.55)	28.57 (27.32;29.81)
Small markets and grocery stores	64.17 (62.45;65.88)	4.70 (4.06;5.32)	31.13 (29.62;32.64)
Fruits, vegetables, and farm products	89.94 (85.54;94.33)	3.72 (1.62;5.80)	6.34 (2.83;6.34)
Bakeries and confectionaries	18.18 (15.41;20.95)	44.29 (40.99;47.59)	37.53 (33.93;41.10)
Butcher shops	85.09 (81.22;88.96)	1.43 (0.23;2.60)	13.48 (9.92;17.03)
Food street vendors	80.96 (75.57;86.36)	4.42 (2.43;6.39)	14.62 (9.40;19.82)
Snack bars	8.79 (2.04;15.56)	0.02 (-0.01;0.04)	91.19 (84.42;97.94)
Restaurants	89.96 (84.26;95.66)	0.29 (-0.27;0.84)	9.75 (4.04;15.44)
Frozen and ready-to-eat food vendors	33.45 (20.21;46.70)	0.17 (-0.15;0.48)	66.38 (52.98;79.75)
Bars	14.29 (2.36;26.22)	8.75 (-0.79;18.28)	76.96 (61.08;92.84)
Convenience stores	17.23 (-1.53;36.01)	6.06 (-1.14;13.20)	76.71 (57.97;95.43)
Fish markets	100.00 (100.00;100.00)	0.00 (0.00;0.00)	0.00 (0.00;0.00)
Candy stores	16.51 (-10.23;43.25)	0.00 (0.00;0.00)	83.49 (80.61;96.46)
Dairy and cold cuts retailers	38.59 (3.23;73.94)	60.19 (24.97;95.42)	1.22 (-0.42;2.85)
Canteens	86.24 (58.25;114.22)	0.00 (0.00;0.00)	13.76 (-14.22;41.74)
Others	53.90 (41.62;66.20)	7.84 (2.12;13.54)	38.26 (26.42;50.07)

Legend:

Source of acquisition of unprocessed or minimally processed foods and processed culinary ingredients (green)

Source of acquisition of processed foods (yellow)

Source of acquisition of ultra-processed foods (red)

Note: [a]G1+G2=unprocessed or minimally processed food (G1) and processed culinary ingredients (G2); [b]G3=processed foods; [c]G4=ultra-processed foods.

Supplementary Table 7. Average percentages (%) and confidence intervals (95%CI) of the share of each Nova Classification food group in total grams acquired at each purchasing place. Midwest Region, Brazil, 2017-2018 (n = 57,920)

Food purchasing places	Average percentage (%) grams		
	G1+G2[a]	G3[b]	G4[c]
	% (95%CI)	% (95%CI)	% (95%CI)
Criteria for classifying food source purchasing places - Midwest	69.37 (66.96;71.77)	8.58 (7.88;9.29)	22.05 (19.92;24.17)
Supermarkets	73.96 (72.76;75.16)	3.15 (2.77;3.53)	22.89 (20.86;24.92)
Small markets and grocery stores	69.30 (65.59;73.01)	4.88 (3.88;5.82)	25.82 (22.25;29.21)
Fruits, vegetables, and farm products	93.10 (90.22;95.96)	2.11 (1.25;2.96)	4.79 (2.16;7.42)
Bakeries and confectionaries	20.44 (16.36;24.53)	49.29 (45.35;53.21)	30.27 (30.26;33.78)
Butcher shops	89.24 (87.26;91;22)	2.57 (1.27;3.84)	8.19 (6.42;9.95)
Food street vendors	81.84 (74.97;88.72)	8.77 (2.62;14.90)	9.39 (4.47;14.29)
Snack bars	6.08 (3.08;9.09)	1.83 (-0.80;4.44)	92.09 (88.15;96.02)
Restaurants	77.54 (66.96;88.09)	0.59 (-0.20;1.28)	21.87 (11.46;32.29)
Frozen and ready-to-eat food vendors	52.76 (25.92;79.62)	0.00 (0.00;0.00)	47.24 (20.37;74.07)
Bars	10.90 (0.08;21.90)	2.19 (-2.00;6.37)	86.91 (75.70;98.10)
Convenience stores	11.17 (0.69;21.62)	10.82 (-4.95;26.61)	78.01 (59.84;96.18)
Fish markets	100.00 (100.00;100.00)	0.00 (0.00;0.00)	0.00 (0.00;0.00)
Candy stores	0.00 (0.00;0.00)	0.00 (0.00;0.00)	100 (100;100)
Dairy and cold cuts retailers	8.52 (-0.25;17.31)	45.06 (27.79;62.32)	46.42 (27.34;65.47)
Canteens	52.17 (4.62;99.72)	0.40 (-0.39;1.19)	47.43 (-0.21;95.16)
Others	53.82 (39.29;68.36)	1.65 (-0.16;3.46)	44.53 (29.90;59.13)

Legend:
Source of acquisition of unprocessed or minimally processed foods and processed culinary ingredients
Source of acquisition of processed foods
Source of acquisition of ultra-processed foods

Note: [a]G1+G2=unprocessed or minimally processed food (G1) and processed culinary ingredients (G2); [b]G3=processed foods; [c]G4=ultra-processed foods.
