# Supplementary material for: Food purchasing places classification system based on the Dietary Guidelines for the Brazilian Population: Locais-Nova
Source: Epidemiol Serv Saude. 2025 Apr 7;34:e20240361. doi: 10.1590/S2237-96222025v34.20240361.en (PMC11998909; doi:10.1590/S2237-96222025v34.20240361.en)
Supplement: Translation

# Translation: Sistema de classificação dos locais de aquisição de alimentos com base no Guia Alimentar para a População Brasileira: Locais-Nova

**DOI:** 10.1590/S2237-96222025v34.20240361.pt

**Authors:** Marcos Anderson Lucas da Silva, Larissa Loures Mendes, Maria Alvim Leite, Luana Lara Rocha, Camila Aparecida Borges, Renata Bertazzi Levy, Maria Laura da Costa Louzada

**Affiliations:** 1 Universidade de São Paulo, Faculdade de Saúde Pública, Programa de Pós-Graduação em Nutrição em Saúde Pública, São Paulo, SP, Brasil; 2 Universidade de São Paulo, Núcleo de Pesquisas Epidemiológicas em Nutrição e Saúde, São Paulo, SP, Brasil; 3 Universidade Federal de Minas Gerais, Escola de Enfermagem, Programa de Pós-graduação em Saúde Pública, Belo Horizonte, MG, Brasil; 4 Universidade Federal de Minas Gerais, Grupo de Estudos, Pesquisas e Práticas em Ambiente Alimentar e Saúde, Belo Horizonte, MG, Brasil; 5 Universidade de São Paulo, Faculdade de Medicina, Departamento de Medicina Preventiva, São Paulo, SP, Brasil; 6 Universidade de São Paulo, Escola Superior de Agricultura Luiz de Queiroz, Departamento de Ciência e Tecnologia de Alimentos, Piracicaba, SP, Brasil

**Keywords:** Guias Alimentares, Atenção Primária, Locais de Aquisição, Classificação Nova, Aquisição de Alimentos

## Abstract

**Objetivo:**

Propor um novo sistema de classificação de locais de aquisição de alimentos com base no *Guia Alimentar para a População Brasileira*, denominado Locais-Nova.

**Métodos:**

Trata-se da utilização dos dados de aquisição domiciliar de alimentos da Pesquisa de Orçamentos Familiares 2017–2018. Os alimentos foram categorizados, segundo a classificação Nova, em alimentos in natura ou minimamente processados, ingredientes culinários, alimentos processados e alimentos ultraprocessados. Estimou-se a contribuição média de cada grupo da classificação Nova para o total de gramas adquiridas no Brasil. Comparou-se essa estimativa com a contribuição média de cada grupo da classificação Nova em cada um dos 16 locais de aquisição avaliados. Os locais foram classificados como “fontes de aquisição” para um grupo específico da classificação Nova sempre que a contribuição desse grupo em determinado local foi igual ou superior à média nacional.

**Resultados:**

A Locais-Nova identificou três categorias de locais de aquisição: fontes de alimentos in natura ou minimamente processados e ingredientes culinários, fontes de alimentos processados e fontes de alimentos ultraprocessados. Hortifrutigranjeiros e açougues destacaram-se como principais fontes de alimentos in natura ou minimamente processados; minimercados e mercearias, como principais fontes de alimentos ultraprocessados; e padarias e confeitarias, como fontes de alimentos processados e ultraprocessados. Supermercados foram classificados como fontes de alimentos in natura ou minimamente processados e ultraprocessados.

**Conclusão:**

Este estudo apresentou a classificação inovadora dos locais de aquisição de alimentos. Isso refletiu as recomendações do *Guia Alimentar para a População Brasileira* e possibilitou entender os padrões de aquisição alimentar nos diferentes tipos de locais.

## Introdução

O *Guia Alimentar para a População Brasileira* foi publicado em 2014 pelo Ministério da Saúde. Ele recomenda que a alimentação seja baseada em alimentos in natura ou minimamente processados e preparações culinárias e que os alimentos ultraprocessados sejam evitados (1). Evidências demonstram que a população vem se afastando dessas recomendações. A participação calórica de alimentos ultraprocessados aumentou de 14,3%, entre 2002–2003, para 19,4%, entre 2017–2018. Nesses mesmos períodos, os alimentos in natura ou minimamente processados passaram de 51,0% para 48,7%, e os ingredientes culinários, de 25,5% para 21,6% (2). 

Existem potenciais conexões entre essa transição dos padrões alimentares e as recentes mudanças nas estruturas do acesso aos alimentos (3), incluindo nos ambientes de varejo. Os locais onde as pessoas adquirem alimentos podem influenciar o consumo alimentar por meio de características como localização e acessibilidade geográfica e variedade, qualidade, preço e estratégias publicitárias dos produtos (4,5). O guia coloca como obstáculos para a alimentação saudável a ubiquidade de locais de aquisição de alimentos que vendem majoritariamente ultraprocessados, usualmente acompanhados de propagandas, e a dificuldade de acesso a locais que vendam alimentos in natura ou minimamente processados, particularmente os perecíveis, como frutas, verduras e legumes (1). 

No Brasil, é possível observar mudanças nos padrões de locais de aquisição de alimentos. Entre 2003 e 2009, a participação dos supermercados no total de compras aumentou de 49,0% (6) para 59,0% (7). Em paralelo, a aquisição em locais mais tradicionais, como feiras livres, tem diminuído (6,7), havendo menor densidade de locais com venda de alimentos saudáveis em alguns territórios do Brasil (8-10).

Os locais de aquisição de alimentos podem ser moldados por políticas públicas e regulamentações que promovam ou restrinjam o acesso a determinados alimentos. Incorporar a análise da dinâmica desses locais nas ações de vigilância alimentar e nutricional pode ser uma estratégia importante para monitorar os obstáculos e facilitadores para uma alimentação saudável. Isso pode contribuir significativamente para a formulação e implementação de ações que visem reduzir as desigualdades no acesso a alimentos de qualidade (11). Na Atenção Primária à Saúde, por exemplo, é cada vez mais recomendado que os profissionais de saúde conheçam os locais de produção, comercialização e distribuição de alimentos, sendo esse conhecimento fundamental para embasar práticas de cuidado em saúde alinhadas à realidade dos indivíduos, comunidades e seus territórios (11).

Nas últimas décadas, foram propostos modelos conceituais e métricas para caracterizar e avaliar a qualidade dos ambientes do varejo (12-18). No Brasil, em 2018, a Câmara Interministerial de Segurança Alimentar e Nutricional apresentou metodologia, de alcance nacional, com objetivo de entender a distribuição territorial do acesso limitado aos alimentos saudáveis no país (19). Algumas fragilidades nessa classificação foram apontadas (9,20,21). Por exemplo, locais de aquisição foram categorizados como “estabelecimentos de aquisição de in natura” ou “estabelecimentos de aquisição de ultraprocessados” se mais de 50,0% dos alimentos adquiridos pertencerem a esses grupos (19), embora não haja fundamento claro para a escolha desse critério. Os “estabelecimentos mistos” foram “onde há predominância de aquisição de preparações culinárias ou alimentos processados ou onde não há predominância de aquisição de alimentos in natura/minimamente processados nem de alimentos ultraprocessados” (19). Esses estabelecimentos incluíam locais que vendiam alimentos saudáveis e não saudáveis, o que dificulta o monitoramento eficaz dos ambientes de compra de alimentos.

Este artigo tem como objetivo propor uma nova classificação dos locais de aquisição de alimentos com base nas recomendações do *Guia Alimentar para a População Brasileira*, denominada Locais-Nova. A proposta visa disponibilizar uma nova referência que seja útil na categorização dos locais de aquisição de alimentos e que apoie a vigilância alimentar e nutricional de determinantes da alimentação saudável.

## Métodos

### Fonte de dados e mensuração

Foram utilizados dados de aquisição domiciliar de alimentos da Pesquisa de Orçamentos Familiares 2017–2018, realizada pelo Instituto Brasileiro de Geografia e Estatística (22) entre julho de 2017 e julho de 2018.

A pesquisa teve abrangência nacional, representativa para os seguintes domínios: o país, as cinco Grandes Regiões (Norte, Nordeste, Centro-Oeste, Sudeste e Sul), as áreas (urbana ou rural), as 26 Unidades da Federação e o Distrito Federal. Utilizou-se plano amostral complexo, por conglomerados em dois estágios, sorteando setores censitários no primeiro estágio e domicílios no segundo. Os setores censitários foram provenientes de amostra mestra do Instituto Brasileiro de Geografia e Estatística, agrupados em estratos de domicílios com alta homogeneidade geográfica e socioeconômica (22).

As informações analisadas referiram-se às aquisições de alimentos para consumo domiciliar feitas pela unidade de consumo (domicílio) durante sete dias consecutivos. Essas aquisições foram registradas pelos moradores do domicílio, ou por um entrevistador do instituto, na caderneta de despesas coletivas, considerando aquisições monetárias e não monetárias, nome do item, quantidade adquirida (quilogramas ou litros) e locais de aquisição dos alimentos (22).

Foram utilizados como unidade de estudo agregados de domicílios gerados no plano amostral (estratos). Em 2017–2018, os 57.920 domicílios estudados resultaram em 575 estratos com a média de 86,50 domicílios (variando de 16 a 524). Neste artigo, foram incluídas somente informações das aquisições monetárias.

### Classificação dos alimentos

Os alimentos foram categorizados segundo a classificação Nova (23). Utilizaram-se como base as recomendações do guia (1), o qual dividiu os alimentos em quatro grupos conforme as características do processamento industrial: alimentos in natura ou minimamente processados (arroz, feijão, legumes e verduras) – G1; ingredientes culinários processados (sal, açúcar) – G2; alimentos processados (queijos, pães) – G3; e alimentos ultraprocessados (refrigerantes, salgadinhos) – G4 (23).

Os grupos de alimentos in natura ou minimamente processados e ingredientes culinários foram avaliados em único grupo (G1+G2). Este agrupamento se alinhou à regra de ouro do guia (“prefira sempre alimentos in natura ou minimamente processados e preparações culinárias a alimentos ultraprocessados”) (1). 

As quantidades de alimentos adquiridos foram expressas como percentual médio da contribuição de cada grupo de alimento para o total de gramas adquiridas. Utilizaram-se fatores de correção para estimar a fração disponível para consumo, excluindo as partes não comestíveis (como cascas ou ossos) (24). As bebidas alcoólicas foram excluídas. 

### Agrupamento dos locais de aquisição de alimentos

Os locais de aquisição de alimentos citados na Pesquisa de Orçamentos Familiares 2017-2018 (n=296) foram agrupados por similaridade com base no estudo técnico de Mapeamento dos Desertos Alimentares no Brasil (19). Este utilizou como referência a Classificação Nacional de Atividades Econômicas, oficialmente adotada por órgãos federais gestores de registros administrativos, para padronizar a classificação dos estabelecimentos conforme a principal atividade (19). Foram criados 16 grupos, selecionando apenas locais de aquisição monetária: supermercados; minimercados e mercearias; hortifrutigranjeiros; padarias e confeitarias; açougues; ambulantes de alimentos; lanchonetes; bares; restaurantes; lojas de conveniência; peixarias; bombonieres; varejistas de laticínios e frios; cantinas de ambientes institucionais; vendas de alimentos congelados e prontos para consumo; e outros (Quadro suplementar 1). 

### Critérios para classificação dos locais de aquisição de alimentos segundo o Guia Alimentar para a População Brasileira: sistema de classificação Locais-Nova

O sistema de classificação Locais-Nova objetiva classificar os locais de aquisição de alimentos segundo o *Guia Alimentar para a População Brasileira*. Foram estabelecidos os critérios (pontos de corte) da Locais-Nova com base no percentual médio de contribuição de cada grupo de alimentos da classificação Nova para o total de gramas adquiridas no país. Estimou-se o percentual médio de contribuição dos grupos de alimentos da classificação Nova para o total de gramas adquiridas em cada local de aquisição. Comparou-se o percentual médio da contribuição de cada grupo de alimentos da classificação Nova em cada local de aquisição com os critérios estabelecidos. Quando o percentual médio de contribuição dos grupos de alimentos, conforme a classificação Nova, em determinado local de aquisição, foi igual ou superior aos critérios definidos, esse local foi classificado como “fonte de aquisição” para aquele grupo de alimentos (Figura 1).

**Figura 1:** Passo-a-passo para a criação dos critérios do sistema de classificação Locais-Nova

Em alguns casos, um local de aquisição pôde ser fonte de dois grupos de alimentos. Isso aconteceu quando o percentual médio da contribuição daquele local foi igual ou superior à média dos critérios para dois grupos de alimentos. 

As três categorias do sistema de classificação Locais-Nova foram: fontes de aquisição de alimentos in natura ou minimamente processados e de ingredientes culinários, fontes de aquisição de alimentos processados e fontes de aquisição de alimentos ultraprocessados. Foram criados critérios, utilizando a mesma metodologia, para as cinco Grandes Regiões do país.

Criaram-se cinco indicadores para identificar os locais de aquisição de alimentos conforme as recomendações do guia.

Locais de aquisição fonte de alimentos que devem ser a base da alimentação: fonte de aquisição de alimentos in natura ou minimamente processados e de ingredientes culinários.
Locais de aquisição identificados apenas como fonte de alimentos que devem ser a base da alimentação: fonte de aquisição apenas de alimentos in natura ou minimamente processados e ingredientes culinários
Locais de aquisição fonte de alimentos que devem ser limitados: fonte de aquisição de alimentos processados.
Locais de aquisição fonte de alimentos que devem ser evitados: fonte de aquisição de alimentos ultraprocessados.
Locais de aquisição identificados apenas como fonte de alimentos que devem ser evitados: fontes de aquisição apenas de alimentos ultraprocessados.

Nos resultados, foram apresentadas as estimativas pontuais (médias) do percentual de participação dos grupos de alimentos em cada local de aquisição no Brasil como um todo e nas Grandes Regiões. Os intervalos de confiança de 95% estão presentes nas tabelas suplementares 2 a 7.

Fatores de ponderação foram aplicados em função da estrutura amostral do banco de dados. Para realizar as análises, foi utilizado o software STATA 18/SE.

## Resultados

No Brasil, os pontos de corte do sistema de classificação Locais-Nova foram: ≥64,65% do total de gramas para os locais fonte de aquisição de alimentos in natura ou minimamente processados e de ingredientes culinários; ≥8,87% para locais fonte de alimentos processados; e ≥26,48% para locais fonte de alimentos ultraprocessados (Figura 2).

**Figura 2:** Critérios para a classificação dos locais de aquisição de alimentos. Brasil e Grandes Regiões, 2017-2018

Nas Grandes Regiões, observaram-se diferenças nos critérios. As regiões com maiores pontos de cortes para alimentos in natura ou minimamente processados e de ingredientes culinários foram a Norte (≥71,63%) e a Centro-Oeste (≥69,37%). O Sudeste apresentou maior ponto de corte para alimentos processados (≥9,21%). O Nordeste apresentou maior ponto de corte maior para alimentos ultraprocessados (≥27,57%) (Figura 2).

No Brasil, 5 dos 16 locais de aquisição foram fontes apenas de alimentos in natura ou minimamente processados e ingredientes culinários (hortifrutigranjeiros, açougues, ambulantes de alimentos, peixarias e restaurantes), 1 local foi fonte apenas de alimentos processados (varejista de laticínios e frios) e 5 foram fontes apenas de alimentos ultraprocessados (minimercados e mercearias, lanchonetes, bombonieres, vendas de alimentos congelados e prontos para consumo, e outros) (Tabela 1). Cinco locais foram fontes de 2 grupos. Entre esses, apenas os supermercados foram fonte tanto de alimentos in natura ou minimamente processados e de ingredientes culinários quanto de alimentos ultraprocessados. Os demais (padarias e confeitarias, bares, lojas de conveniência e cantinas) foram fontes de alimentos processados e de ultraprocessados (Tabela 1). 

**Tabela 1:** Percentual (%) médio de contribuição de cada grupo de alimentos da classificação Nova para o total de gramas adquiridas em cada local de aquisição. Brasil, 2017-2018 (n=57.920)

Locais de aquisição de alimentos	G1+G2^a^	G3^b^	G4^c^
**Critérios para classificação dos locais fontes de aquisição de alimentos** - Brasil	≥64,65	≥8,87	≥26,48
Supermercados	69,29	3,67	27,04
Minimercados e mercearias	64,49	4,93	30,58
Hortifrutigranjeiros	93,69	1,81	4,50
Padarias e confeitarias	18,28	54,90	26,82
Açougues	87,75	2,65	9,60
Ambulantes de alimentos	74,76	8,72	16,52
Lanchonetes	11,57	0,89	87,54
Restaurantes	84,08	0,81	15,11
Vendas de alimentos congelados e prontos para consumo	43,23	2,77	54,00
Bares	26,17	9,37	64,46
Lojas de conveniência	7,32	13,63	79,05
Peixarias	99,65	0,35	0,00
Bombonieres	9,54	3,80	86,66
Varejistas de laticínios e frios	32,12	49,05	18,83
Cantinas	44,96	11,62	43,42
Outros	46,74	3,53	49,73

**Legenda:** Local fonte de aquisição de alimentos in natura ou minimamente processados e ingredientes culinários Local fonte de aquisição de alimentos processados Local fonte de aquisição de alimentos ultraprocessados ^a^G1+G2=in natura ou minimamente processados (G1) e ingredientes culinários (G2); ^b^G3=alimentos processados; ^c^G4=alimentos ultraprocessados.

Em todas as regiões, os locais fontes de alimentos in natura ou minimamente processados e de ingredientes culinários foram supermercados, hortifrutigranjeiros, açougues, ambulantes de alimentos, restaurantes e peixarias (Tabela 2). Para os locais fonte de alimentos processados, a diversidade regional foi evidente. No Sul, havia menos locais fontes desse grupo de alimentos (padarias e confeitarias e varejista de laticínios e frios). O Norte, padarias e confeitarias, bares e varejista de laticínios e frios. No Nordeste, como esses locais destacados no Norte, também foram incluídas as cantinas. No Sudeste, foram padarias e confeitarias, ambulantes de alimentos, bares, lojas de conveniência, varejistas de laticínios e frios, e cantinas. No Centro-Oeste, foram padarias e confeitarias, ambulantes de alimentos, lojas de conveniência e varejistas de laticínios e frios (Tabela 2). 

**Tabela 2:** Percentual (%) médio de contribuição de cada grupo de alimentos da classificação Nova para o total de gramas adquiridas em cada local de aquisição segundo as Grandes Regiões do Brasil. 2017-2018 (n=57.920)

Locais de aquisição de alimentos	Norte			Nordeste			Sudeste			Sul			Centro-Oeste		
G1+G2^a^	G3^b^	G4^c^	G1+G2^a^	G3^b^	G4^c^	G1+G2^a^	G3^b^	G4^c^	G1+G2^a^	G3^b^	G4^c^	G1+G2^a^	G3^b^	G4^c^
**Critérios para classificação dos locais fontes de aquisição de alimentos** – **por região**	≥71,63	≥7,60	≥20,77	≥64,22	≥8.21	≥27,57	≥63,07	≥9,41	≥27,52	≥64,21	≥9,21	≥26,58	≥69,37	≥8,58	≥22,05
Supermercados	75,73	3,18	21,09	72,70	3,58	23,72	67,77	3,75	28,48	67,12	4,31	28,57	73,96	3,15	22,89
Minimercados e mercearias	71,53	3,55	24,92	63,22	4,42	32,36	62,30	5,47	32,23	64,17	4,70	31,13	69,30	4,88	25,82
Hortifrutigranjeiros	95,81	1,24	2,95	96,23	1,59	2,18	93,23	1,29	5,48	89,94	3,72	6,34	93,10	2,11	4,79
Padarias e confeitarias	11,39	66,76	21,85	14,49	67,20	18,31	23,63	50,43	25,94	18,18	44,29	37,53	20,44	49,29	30,27
Açougues	95,87	1,51	2,60	92,58	3,94	3,48	84,09	2,43	13,47	85,09	1,42	13,48	89,24	2,57	8,19
Ambulantes de alimentos	80,86	3,65	15,49	75,51	9,29	15,20	69,96	10,69	19,35	80,96	4,42	14,62	81,84	8,77	9,39
Lanchonetes	35,38	0,59	64,03	11,86	2,09	86,05	9,72	0,43	89,85	8,79	0,02	91,19	6,08	1,83	92,09
Restaurantes	90,19	0,71	9,10	86,11	0,99	12,90	81,07	0,96	17,97	89,96	0,29	9,75	77,53	0,59	21,87
Vendas de alimentos congelados e prontos para consumo	46,01	0,00	53,99	29,50	7,27	63,23	51,86	1,95	46,19	33,45	0,17	66,38	52,76	0,00	47,24
Bares	52,06	11,76	36,18	30,49	4,63	64,88	24,10	12,03	63,87	14,29	8,75	76,96	10,90	2,19	86,91
Lojas de conveniência	0,00	0,17	99,83	13,32	7,05	79,63	1,28	21,39	77,33	17,23	6,06	76,71	11,17	10,82	78,01
Peixarias	100,00	0,00	0,00	98,88	1,12	0,00	100,00	0,00	0,00	100,00	0,00	0,00	100,00	0,00	0,00
*Bombonieres*	0,00	4,19	95,81	16,65	6,86	76,49	7,42	4,05	88,53	16,51	0,00	83,49	0,00	0,00	100
Varejistas de laticínios e frios	34,85	31,66	33,49	49,32	38,15	12,53	21,02	54,46	24,52	38,59	60,19	1,22	8,52	45,06	46,42
Cantinas	56,02	0,00	43,98	58,65	8,38	32,97	22,87	20,66	56,47	86,24	0,00	13,76	52,17	0,40	47,43
Outros	49,07	1,68	49,25	57,42	3,51	39,07	36,78	2,59	60,63	59,90	7,84	38,26	53,82	1,65	44,53

**Legenda:** Local fonte de aquisição de alimentos in natura ou minimamente processados e ingredientes culinários Local fonte de aquisição de alimentos processados Local fonte de aquisição de alimentos ultraprocessados ^a^G1+G2=in natura ou minimamente processados (G1) e ingredientes culinários (G2); ^b^G3=alimentos processados; ^c^G4=alimentos ultraprocessados.

Em todas as regiões, supermercados, minimercados e mercearias, padarias e confeitarias, lanchonetes, bares, lojas de conveniência, bombonieres, vendas de alimentos congelados e prontos para consumo e outros foram fontes de ultraprocessados. No Norte e no Centro-Oeste, varejistas de laticínios e frios também foram incluídos nessa categoria (Tabela 2).

Embora todos os locais tenham sido classificados segundo os critérios do Locais-Nova com base nas médias estimadas, locais com baixa contribuição nas aquisições, como cantinas no Brasil todo, bares e varejistas de laticínios e frios no Norte, cantinas no Nordeste, e bares e lojas de conveniência no Sudeste e Centro-Oeste, apresentaram intervalos de confiança amplos (IC95%), tornando sua classificação incerta. (tabela suplementar 2)

Hortifrutigranjeiros, açougues, restaurantes e peixarias apareceram em todas as regiões do país como locais de aquisição exclusivamente fontes de alimentos que devem ser a base da alimentação (Quadro 1). O Nordeste foi a única região que incluiu supermercados nessa categoria, enquanto no Sul, cantinas foram incluídas. Minimercados e mercearias, lanchonetes e bombonieres apareceram em todas as regiões quanto aos locais que são exclusivamente fontes de alimentos que devem ser evitados. No Norte e no Centro-Oeste, apareceram as cantinas. No Nordeste, apareceram os bares (Quadro 1). 

**Quadro 1:** Locais de aquisição de alimentos conforme as recomendações do Guia Alimentar para a População Brasileira. Brasil e Grandes Regiões, 2017-2018 (n=57.920)

Indicadores	Brasil	Norte	Nordeste	Sudeste	Sul	Centro-Oeste
Locais de aquisição fonte de alimentos que devem ser a base da alimentação - fonte de aquisição de alimentos in natura ou minimamente processados e de ingredientes culinários	Supermercados Hortifrutigranjeiros Açougues Ambulantes de alimentos Restaurantes Peixarias	Supermercados Hortifrutigranjeiros Açougues Ambulantes de alimentos Restaurantes Peixarias	Supermercados Hortifrutigranjeiros Açougues Ambulantes de alimentos Restaurantes Peixarias	Supermercados Hortifrutigranjeiros Açougues Ambulantes de alimentos Restaurantes Peixarias	Supermercados Hortifrutigranjeiros Açougues Ambulantes de alimentos Restaurantes Peixarias Cantinas	Supermercados Hortifrutigranjeiros Açougues Ambulantes de alimentos Restaurantes Peixarias
Locais de aquisição identificados apenas como fonte de alimentos que devem ser a base da alimentação - fonte de aquisição apenas de alimentos in natura ou minimamente processados e ingredientes culinários	Hortifrutigranjeiros Açougues Ambulantes de alimentos Peixarias Restaurantes	Hortifrutigranjeiros Açougues Ambulantes de alimentos Restaurantes Peixarias	Supermercados Hortifrutigranjeiros Açougues Restaurantes Peixarias	Hortifrutigranjeiros Açougues Restaurantes Peixarias	Hortifrutigranjeiros Açougues Ambulantes de alimentos Peixarias Restaurantes Cantinas	Hortifrutigranjeiros Açougues Restaurantes Peixarias
Locais de aquisição fonte de alimentos que devem ser limitados - fonte de aquisição de alimentos processados	Padarias e confeitarias Bares Lojas de conveniência Varejistas de laticínios e frios Cantinas	Padarias e confeitarias Bares Varejistas de laticínios e frios	Padarias e confeitarias Ambulantes de alimentação Varejistas de laticínios e frios Cantinas	Padarias e confeitarias Ambulantes de alimentação Bares Lojas de conveniência Varejistas de laticínios e frios cantinas	Padarias e confeitarias Varejistas de laticínios e frios	Padarias e confeitarias Ambulantes de alimentos Lojas de conveniência Varejistas de laticínios e frios
Locais de aquisição fonte de alimentos que devem ser evitados - fonte de aquisição de alimentos ultraprocessados	Supermercados Minimercados e mercearias Padarias e confeitarias Lanchonetes Bares Lojas de conveniência Bombonieres Cantinas Vendas de alimentos congelados e prontos para consumo Outros	Supermercados Minimercados e mercearias Padarias e confeitarias Lanchonetes Bares Lojas de conveniência Bombonieres Varejista de laticínios e frios Cantinas Vendas de alimentos congelados e prontos para consumo Outros	Minimercados e mercearias Lanchonetes Bares Lojas de conveniência Bombonieres Cantinas Vendas de alimentos congelados e prontos para consumo Outros	Supermercados Minimercados e mercearias Lanchonetes Bares Lojas de conveniência Bombonieres Cantinas Vendas de alimentos congelados e prontos para consumo Outros	Supermercados Minimercados e mercearias Padarias e confeitarias Lanchonetes Bares Lojas de conveniência Bombonieres Vendas de alimentos congelados e prontos para consumo Outros	Supermercados Minimercados e mercearias Padarias e confeitarias Lanchonetes Bares Lojas de conveniência Bombonieres Varejistas de laticínios e frios Cantinas Vendas de alimentos congelados e prontos para consumo Outros
Locais de aquisição identificados apenas como fonte de alimentos que devem ser evitados - fontes de aquisição apenas de alimentos ultraprocessados	Minimercados e mercearias Lanchonetes Bombonieres Vendas de alimentos congelados e prontos para consumo Outros	Minimercados e mercearias Lanchonetes Lojas de conveniência Bombonieres Cantinas Vendas de alimentos congelados e prontos para consumo Outros	Minimercados e mercearias Lanchonetes Bares Lojas de conveniência Bombonieres Vendas de alimentos congelados e prontos para consumo Outros	Minimercados e mercearias Lanchonetes Bombonieres Vendas de alimentos congelados e prontos para consumo Outros	Minimercados e mercearias Lanchonetes Bares Bombonieres Vendas de alimentos congelados e prontos para consumo Outros	Minimercados e mercearias Lanchonetes Bares Bombonieres Cantinas Vendas de alimentos congelados e prontos para consumo Outros

## Discussão

Este estudo apresentou uma nova metodologia para classificação dos locais de aquisição de alimentos segundo o guia alimentar. Ao analisar grupos de alimentos adquiridos em diferentes categorias de locais, foram estabelecidos critérios capazes de distinguir entre locais que promovem a alimentação saudável e locais que incentivam a não saudável, considerando o padrão de aquisição nos variados locais do Brasil. A estratificação regional permitiu customizar essa classificação para contextos geográficos específicos, oferecendo mais detalhes sobre os ambientes alimentares. 

As particularidades econômicas e culturais de cada região influenciaram nas características dos ambientes e nas dinâmicas de acesso aos alimentos (2,25). Isso explicou o porquê de um local de aquisição ser fonte de um grupo de alimentos em uma região, mas não em outra. Considerando os locais que, cumulativamente, contribuíram com mais de 90% do total adquirido (Tabela suplementar 1), notou-se que hortifrutigranjeiros e açougues foram consistentemente identificados como fontes de alimentos que devem ser a base da alimentação brasileira. Minimercados e mercearias foram classificados como locais a serem evitados. O supermercado foi o único local identificado como fonte de in natura ou minimamente processados e ingredientes culinários e de ultraprocessados.

Os dados de aquisição de alimentos mostraram que os supermercados foram os locais de aquisição de alimentos mais acessados no Brasil, seguidos por pequenos mercados, feiras livres e padarias (6,7). Embora o supermercado seja uma fonte de alimentos saudáveis, outras evidências indicaram que utilizá-lo como a única fonte de aquisição de alimentos saudáveis pode não ser a estratégia mais adequada (26,27). Isso porque os alimentos in natura ou minimamente processados frequentemente competem com a abundância de alimentos ultraprocessados nesse local de aquisição, como refrigerantes, biscoitos, entre outros (1). Dentro destes locais, esses alimentos são frequentemente promovidos por meio de propaganda agressiva e promoções atraentes (5,28,29), o que pode prejudicar escolhas alimentares mais saudáveis.

Os supermercados são considerados mais convenientes e, na aquisição geral, apresentam preços em média 37% menores em comparação com outros locais de aquisição (30). A proximidade entre esses locais das feiras livres pode ter impacto negativo. Evidências mostraram que a presença de supermercados próximos a esses locais levou à redução dos preços de frutas, legumes e verduras nos supermercados, em uma tentativa de competir com as feiras livres das proximidades (31). Se o número de feiras livres diminuir, os supermercados podem aumentar os preços devido à ausência de concorrência, dificultando o acesso a alimentos saudáveis e diversificados para a população.

Foi criado também o indicador “Locais de aquisição identificado apenas como fonte de alimentos que devem ser a base da alimentação”, que inclui locais que, utilizando o método proposto, foram considerados fonte de alimentos in natura ou minimamente processados e de ingredientes culinários. Esse indicador foi proposto como a métrica que identifica locais com grupos de alimentos que devem ser a base da alimentação. Hortifrutigranjeiros, açougues, peixarias e restaurantes, identificados como locais de aquisição de alimentos que devem ser a base da alimentação, têm o potencial de desempenhar papel crucial na promoção da alimentação saudável. 

A metodologia apresentada neste artigo possibilita o mapeamento do acesso a alimentos no Brasil com grande abrangência e escala. Isso permitiu observar a especificidade do local, onde a realização de auditorias detalhadas seria inviável. Essa classificação pode ser aplicada, por exemplo, em bases de dados de registros de coordenadas geográficas de locais de vendas de alimentos. Um exemplo dessas bases é a Relação Anual de Informações Sociais, uma base de dados governamental de acesso aberto. Nela, os locais de aquisição são classificados segundo a Classificação Nacional de Atividades Econômicas, o que permite a identificação de diferentes locais de aquisição de alimentos (32).

A aplicação regular do sistema de classificação Locais-Nova a dados de distribuição de locais de aquisição de alimentos permitirá identificar e monitorar as transformações desses locais. Novos padrões de aquisição poderão emergir e determinados locais poderão se tornar fontes de alimentos diferentes ou permanecer como estão. Esse monitoramento permitirá o desenvolvimento de estratégias para evitar que os locais se tornem predominantemente fonte de alimentos ultraprocessados e a priorização de territórios que necessitam de equipamentos que ofereçam alimentos saudáveis. Entre as possíveis medidas, destacam-se incentivos financeiros ou fiscais para comerciantes que aumentarem a oferta de alimentos saudáveis, como frutas, legumes, verduras e outros alimentos in natura ou minimamente processados. Destaca-se também a criação de novos pontos de venda, como feiras ou mercados públicos, especialmente em áreas com baixa disponibilidade de alimentos saudáveis. 

Atualizações contínuas do sistema de classificação apresentado neste artigo poderão, na existência de fontes de informação, incorporar indicadores que contemplem a avaliação de características adicionais dos locais, como preço, publicidade e porte dos locais de aquisição de alimentos. A Locais-Nova é passível de ser reproduzida em outros países e contextos em que estejam disponíveis bases de dados sobre aquisição de alimentos, gerando pontos de corte específicos de cada território.

Houve algumas limitações, incluindo o fato de o inquérito de aquisição da Pesquisa de Orçamentos Familiares não considerar a aquisição de alimentos para consumo fora do domicílio. Isso prejudica, em particular, a avaliação de estabelecimentos como cantinas, restaurantes, bares, lanchonetes, ambulantes de alimentos e varejistas de laticínios e frios, que juntos representaram menos de 1% na participação da aquisição total. Apesar disso, a maioria das aquisições foi para consumo nos domicílios (33). Os dados do inquérito foram de 2017-2018 e o cenário pode ter mudado significativamente, especialmente no período pós-pandemia de covid-19, com o aumento das compras online (34,35). 

A Pesquisa de Orçamentos Familiares 2017–2018 é o inquérito nacional mais recente com representatividade nacional e possui nível de detalhamento suficiente para identificar os locais de aquisição e grupos de alimentos estudados. A metodologia poderá ser aplicada à nova pesquisa assim que ela for publicada. O uso da classificação Nova proporciona a análise dos locais de compra com base no grau de processamento dos alimentos, paradigma crucial para compreender sistemas alimentares atualmente. Este estudo também apresentou a metodologia alinhada ao escopo da vigilância alimentar e nutricional e direcionada às recomendações do guia. 

Em conclusão, foi apresentado novo sistema de classificação para os locais de aquisição de alimentos, fundamentado nas diretrizes do *Guia Alimentar para a População Brasileira*, para identificar como diferentes composições dos ambientes alimentares influenciam os padrões de consumo. 

As disparidades regionais sugerem a necessidade de estratégias adaptadas às especificidades locais. A inclusão dessa metodologia em futuros inquéritos possibilitará o monitoramento contínuo e detalhado do ambiente alimentar, apoiando a formulação de políticas públicas que visem melhorar a saúde da população brasileira, incidindo no varejo de alimentos.
